# Comparative Proteomic and Physiological Analysis Reveals the Variation Mechanisms of Leaf Coloration and Carbon Fixation in a Xantha Mutant of *Ginkgo biloba* L.

**DOI:** 10.3390/ijms17111794

**Published:** 2016-10-27

**Authors:** Xinliang Liu, Wanwen Yu, Guibin Wang, Fuliang Cao, Jinfeng Cai, Huanli Wang

**Affiliations:** 1 Co-Innovation Center for Sustainable Forestry in Southern China, Nanjing Forestry University, Nanjing 210037, China; xliu25@lakeheadu.ca (X.L.); youeryuww@163.com (W.Y.); gbwang@njfu.edu.cn (G.W.); caijinfeng1984@126.com (J.C.); 2 The Jiangsu Provincial Platform for Conservation and Utilization of Agricultural Germplasm, Nanjing 210037, China; 3 Institute of Botany, Jiangsu Province and Chinese Academy of Sciences, Nanjing 210037, China; wanghuanlis@163.com

**Keywords:** *Ginkgo biloba* L., xantha mutant, comparative proteomics, chloroplast, photosynthesis

## Abstract

Yellow-green leaf mutants are common in higher plants, and these non-lethal chlorophyll-deficient mutants are ideal materials for research on photosynthesis and plant development. A novel xantha mutant of *Ginkgo biloba* displaying yellow-colour leaves (YL) and green-colour leaves (GL) was identified in this study. The chlorophyll content of YL was remarkably lower than that in GL. The chloroplast ultrastructure revealed that YL had less dense thylakoid lamellae, a looser structure and fewer starch grains than GL. Analysis of the photosynthetic characteristics revealed that YL had decreased photosynthetic activity with significantly high nonphotochemical quenching. To explain these phenomena, we analysed the proteomic differences in leaves and chloroplasts between YL and GL of ginkgo using two-dimensional gel electrophoresis (2-DE) coupled with MALDI-TOF/TOF MS. In total, 89 differential proteins were successfully identified, 82 of which were assigned functions in nine metabolic pathways and cellular processes. Among them, proteins involved in photosynthesis, carbon fixation in photosynthetic organisms, carbohydrate/energy metabolism, amino acid metabolism, and protein metabolism were greatly enriched, indicating a good correlation between differentially accumulated proteins and physiological changes in leaves. The identifications of these differentially accumulated proteins indicates the presence of a specific different metabolic network in YL and suggests that YL possess slower chloroplast development, weaker photosynthesis, and a less abundant energy supply than GL. These studies provide insights into the mechanism of molecular regulation of leaf colour variation in YL mutants.

## 1. Introduction

Chlorophyll (Chl) is the most important pigment in plants and is usually embedded in the thylakoid membranes of chloroplasts [1,2]. Chl is a green pigment, essential for photosynthesis, that absorbs energy from sunlight in antenna systems and transfers the energy to the reaction centre [3]. The absorbed light energy is then used to synthesize carbohydrates from carbon dioxide and water, a fundamental life process in plants. In higher plants, Chl is mainly biosynthesized in plastids, and its metabolic pathway has been extensively studied using genetic and biochemical methods in various organisms, particularly *Arabidopsis thaliana* [4,5,6]. Mutations in Chl biosynthesis, degradation or other related pathways lead to Chl-deficient mutants or leaf colour mutants. These mutants are widespread in nature and yield various mutant leaf colours, such as albino, virescent, chlorina, xanthas, maculate, stripe and dark green [7,8,9]. A number of yellow-green leaf colour mutants have been identified in model plants, including *A. thaliana*, *Oryza sativa*, *Solanum lycopersicum*, *Zea mays* and *Triticum aestivum* [10,11,12,13,14].

Yellow-green leaf colour mutants are induced by multiple genetic and environmental factors, among which genetic change plays a decisive role. In *O. sativa*, mutations in the genes that encode glutamyl-tRNA, Mg-chelatase, heme oxygenase and geranylgeranyl reductase lead to yellow-green leaves, whereas T-DNA insertional mutagenesis in rice can generate a chlorina phenotype [7,15,16,17,18]. Gene mutations involved in chloroplast development can also affect leaf colour. In *Arabidopsis*, two leaf-variegated mutants, *yellow variegated1* (*var1*) and *var2*, are caused by the loss of FtsH5 and FtsH2 (ATP-dependent zinc metalloprotease), respectively; these proteins are involved in the repair of photo-damaged proteins in thylakoid membranes [19,20]. The genetic changes in mutants can lead to changes in leaf phenotype, microstructures, fluorescence and physiological properties. These leaf colour mutants are thus ideal materials to study photosynthesis, photomorphogenesis, Chl metabolism, and chloroplast development [13,17,21].

Proteomics analysis deals directly with large-scale changes in proteins, the main components of physiological metabolic pathways in living cells. Proteins are responsible for most biological processes. Proteomics is a powerful approach for investigating the complete proteome at specific development stages or identifying changes in the proteome under different environmental conditions [22]. Proteome analysis, using two-dimensional polyacrylamide gel electrophoresis (2-DE), is one of the most sensitive and potent methods in proteomics studies [23]. Subcellular proteomic analysis has been widely studied in plants, including for the characterization of the network of cellular processes in a particular organelle, such as mitochondria, chloroplasts, nuclei, and plasma membranes [24,25,26]. Chloroplasts are the site of photosynthesis in plants and the largest metabolically active compartments in mature leaves. In *A. thaliana*, proteomics approaches have revealed 73% of unique organelle stress-responsive proteins belong to chloroplasts, indicating the specific sensitivity of chloroplasts to environmental stress [27]. Many proteomic studies have focused on chloroplasts or chloroplast fractions, providing significant insight into chlorophyll metabolism, photosynthesis, or the chloroplast response to environmental stress [25,26,27,28]. A proteomic analysis of the thylakoid membrane of a Chl b-less rice mutant and wild type by Chen et al. suggested that the reduction of Chl b affects light-harvesting complex I (LHC-I) assembly more severely than LHC-II [28]. Wang et al. performed transcriptomic and proteomic analyses of a Chl-deficient tea plant cultivar and suggested a complementary approach to better understand the mechanisms responsible for the chlorina phenotype [29]. Zhou et al. identified two colour patterns of flower buds in an ornamental peach and studied the differential expression of proteins, which provided important insights into the molecular mechanism of flower petal coloration [30]. Therefore, the analysis of the differential expression of proteins between mutant and normal tissue, particularly at the subcellular level, may provide new insights into the regulatory mechanism responsible for the mutant phenotype.

*Ginkgo biloba*, often called a “living fossil”, is the only remaining species of *Ginkgoaceae* and is well-known for its medicinal value and ornamental beauty [31]. Ginkgo is planted throughout China as a multi-value deciduous tree species of ornamental due to its unique leaf pattern and tree form. Recently, we discovered a pigment-deficient mutant of *G. biloba* that exhibited a yellow-green leaf phenotype on a main branch and was initially identified as a xantha mutant in Jiujiang City, Jiangxi Province, China (29°49′ N, 116°40′ E). The mutant is an ancient tree with an estimated age of 150 years, a height of 18.8 m, and a diameter of 1.6 m at 2 m above ground. The branch is supposed to be a bud mutation and constitutes one-fourth of the crown of the tree, with the rest of the tree having green leaves. During the early growth stages, leaves of the xantha mutant are yellow and are remarkably different from green leaves until early July. As the mutant leaves mature, the colour gradually turns yellow-green until October, and finally the leaves turn yellow again. This type of bright and stable leaf colour phenotype is rare in ginkgo, and this mutation is considered a better ornamental germplasm resource for cultivation than wild type. At present, little is known regarding the molecular basis of this leaf mutant.

In this study, we used a proteomic approach to compare the total leaf protein and chloroplast protein profiles of the yellow-colour leaf (YL) and the green-colour leaf (GL) of *G. biloba*. Using a 2-DE proteomic approach and MALDI-TOF/TOF MS analysis, we successfully identified 89 differential proteins, which were predicted to play potential roles in particular cellular processes in the YL mutant. To elucidate the relationship between the variation of photosynthesis and specific proteins with altered expression, we performed photosynthesis characterization and chloroplast ultrastructure observation, leading to a further understanding of the molecular mechanisms of pigment biosynthesis and photosynthesis alteration in the YL mutant.

## 2. Results

### 2.1. Chlorophyll Concentration, Chloroplast Ultrastructure and Photosynthesis Performance

**Figure 1:**
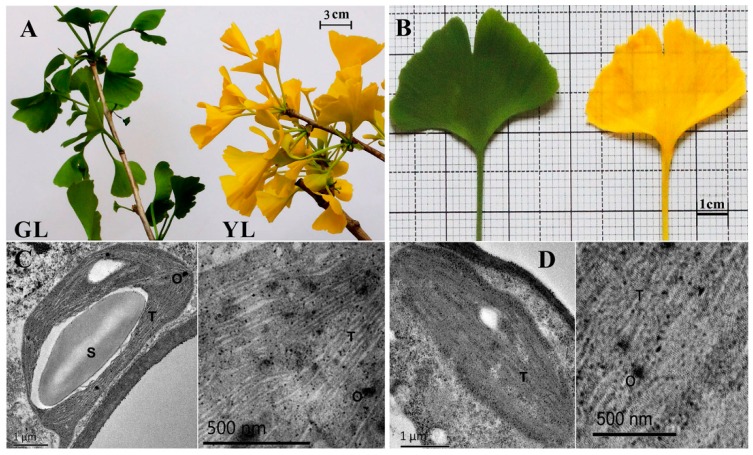

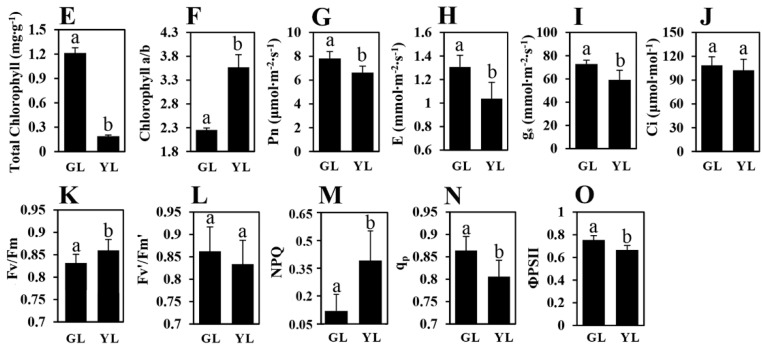
Photosynthetic parameters and chloroplast ultrastructure of ginkgo yellow-colour leaves (YL) and green-colour leaves (GL). (**A**,**B**) Phenotypes of the GL (left) and YL mutant (right); (**C**,**D**) Chloroplast ultrastructure in GL (**C**) and YL (**D**). S, starch grain; T, thylakoid; O, osmiophilic granule; (**E**,**F**) Total chlorophyll content and chlorophyll a/b; (**G**–**J**) Gas exchange parameters in P_n_ (**G**), E (**H**), g_s_ (**I**), C_i_ (**J**); (**K**–**O**) Changes in chlorophyll fluorescence parameters, including maximum quantum efficiency of photosystem II (PSII) (Fv/Fm) (**K**), efficiency of excitation energy capture by open PSII centres (Fv′/Fm′) (**L**), nonphotochemical quenching (NPQ) (**M**), photochemical quenching (q_p_) (**N**), effective quantum yield of PSII electron transport (ΦPSII) (**O**). The values are presented as means ± SD (*n* = 15). Different letters indicate significant differences at *p* < 0.05.

The leaf morphologies of GL and YL are shown in Figure 1A,B. No difference in phenotype was observed between YL and GL, except leaf chlorosis in YL. The chlorophyll a and b contents were markedly higher in GL than in YL, but the chlorophyll a/b ratio was significantly lower in GL than in YL (*p* < 0.01). The gas exchange parameters of the leaves of two colours are shown in Figure 1G–J, and the net photosynthetic rate (P_n_), transportation rate (E) and stomatal conductance (g_s_) were significantly higher in GL than YL (*p* < 0.01). There were no significant differences in the internal CO_2_ concentration (C_i_) between the two types of leaves. As shown in Figure 1K–O, the effective quantum yield of photosystem II electron transport (ΦPSII) and photochemical quenching (q_p_) were significantly higher in GL than in YL (*p* < 0.05). The patterns of the efficiency of excitation energy capture by open photosystem II reaction centres (Fv′/Fm′) was similar in GL and YL, whereas the maximum quantum yield of photosystem II (Fv/Fm) and the nonphotochemical quenching (NPQ) were considerably lower in GL than in YL (*p* < 0.01).

To identify morphological changes in photosynthetic organelles and leaves of GL and YL, the chloroplast ultrastructure was examined. As shown in Figure 1C, the chloroplasts of GL exhibited an elliptical shape, with a typical lamellar grana structure consisting of thylakoid and numerous starch grains and few osmiophilic granules. By contrast, the chloroplasts in YL leaves were abnormal in appearance, with a slim spindle shape with no obvious boundary and simple distributed grana structure, and usually contained no well-defined starch grains and a few small plastoglobules. YL had a reduced number of thylakoids in grana and contained diffuse internal membranes and a dilated lamellae system compared to GL (Figure 1D). In addition, the chloroplasts were smaller in size in YL than in GL.

### 2.2. 2-DE Analysis and Identification of Differentially Accumulated Proteins

**Figure 2:**
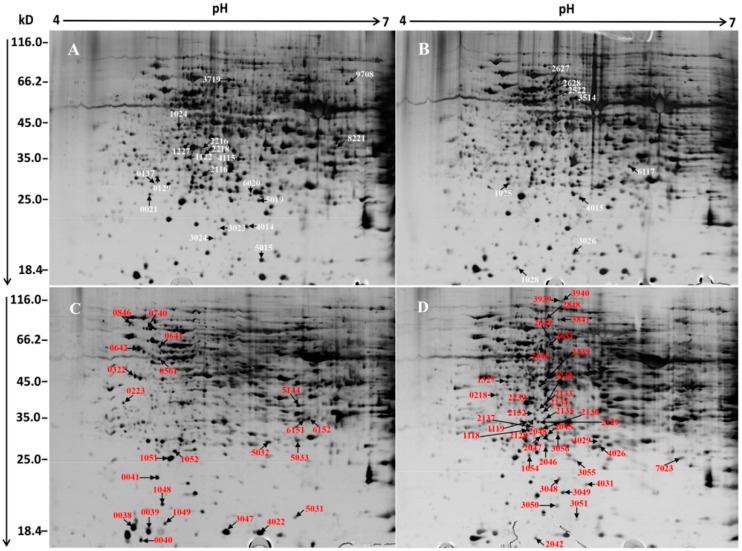
Representative 2-DE profiles of proteins in *G. biloba*. The molecular mass (M_r_) in kDa and pI of proteins are indicated on the left and top, respectively. The identified proteins are annotated by a number and arrow. (**A**) Total proteins from YL; (**B**) Total proteins from GL; (**C**) Chloroplast proteins from YL; (**D**) Chloroplast proteins from GL. The arrows indicate increased protein spots in ginkgo.

To analyse and compare the changes in protein profiles, comparative proteomics of ginkgo leaves and chloroplasts of the two leaves of different colours were performed using 2-DE and MALDI-TOF/TOF MS. The representative 2-DE maps with immobilized pH gradient (IPG) strips of pH 4–7 and 3–10 are shown in Figure 2 and Figure S1 respectively. As more proteins are detectable clearly in the IPG 4–7 gel than in the IPG 3–10 gel, IPG 4–7 gel were used in this study. The 2-DE profile of the total protein in the leaves demonstrated that more than 1300 protein spots were detected with good reproducibility in the range of molecular masses of 15–120 kDa and pH 4–7. A total of 28 protein spots in YL exhibited significant changes in abundance of less than 0.66-fold or more than 1.5-fold (*p* < 0.05) compared to GL (Figure 2A,B). Similar protein patterns were revealed in the 2-DE profile of total leaf proteins and chloroplast proteins. The 2-DE profile of chloroplast proteins revealed that more than 1100 protein spots were detected, and a total of 61 protein spots exhibited significant changes in abundance (Figure 2C,D). After analysis by MALDI-TOF/TOF MS, a total of 89 differentially accumulated protein spots were successfully identified (Table 1). Compared to GL, 42 (47%) of the differential protein spots identified were increased in YL, whereas 47 (53%) were decreased. Sub-cellular localization, predicted by WolfPsort, revealed that the majority of identified proteins were located in the cytoplasm and chloroplast. More than 46% of the identified total leaf proteins were predicted to be located in the chloroplasts, implying that chloroplast proteome is virtually changed in YL. Whereas in the chloroplast, nearly 82% of the identified proteins from chloroplast were predicted to be typical chloroplast proteins.

### 2.3. Functional Categorization of Identified Proteins

**Figure 3:**
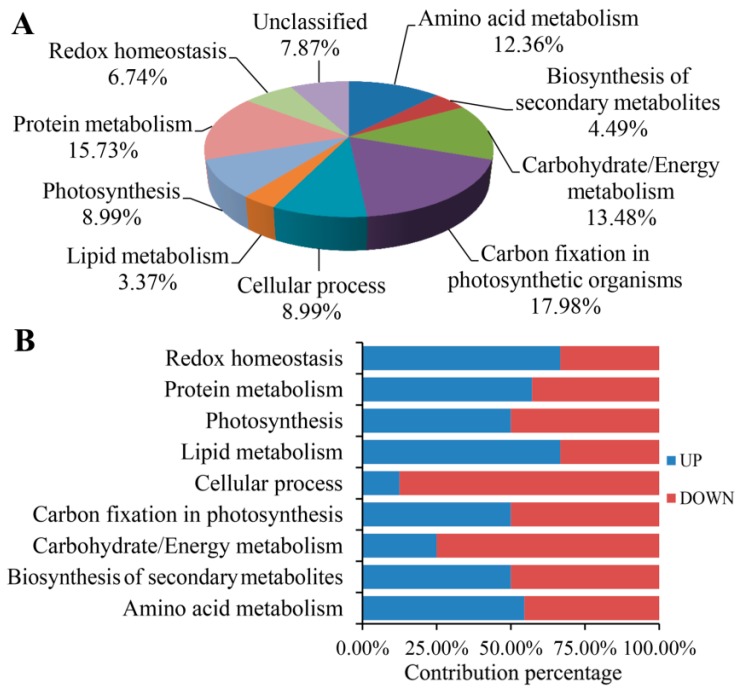
Function classification of all identified proteins in *G. biloba*. (**A**) Functional categorization of identified proteins; (**B**) Contributions to molecular functions from increased (blue) and decreased (red) proteins.

The functions of the identified proteins were annotated by searching against the NCBI database and grouped according to functional categories based on analysis of KEGG pathways and the literature. Among the 89 identified proteins, 82 proteins (92%) had assigned functions or sequences similar to those of known proteins, and the other seven (8%) were identified as novel with no assigned function (Figure 3). The 82 proteins with assigned functions in both types of leaves were classified into nine metabolic pathways and cellular processes, including amino acid metabolism (11 proteins), biosynthesis of secondary metabolites (4 proteins), carbohydrate/energy metabolism (12 proteins), carbon fixation in photosynthetic organisms (16 proteins), cellular processes (8 proteins), lipid metabolism (3 proteins), photosynthesis (8 proteins), protein metabolism (14 proteins), and redox homeostasis (6 proteins) (Figure 3A). The distribution of increased and decreased proteins in the different functional groups is shown in Figure 3B.

### 2.4. Protein-Protein Interaction Analysis

**Figure 4:**
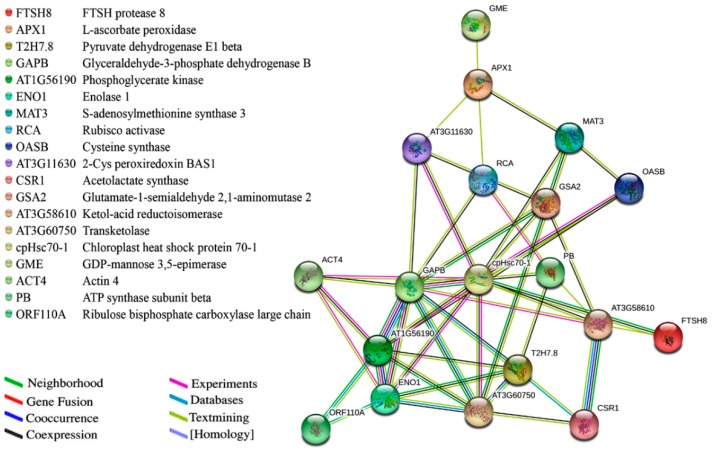
Protein-protein interaction network of differentially accumulated proteins analysed by STRING 10.

To further understand the functional associations of the identified proteins from both types of leaves, a protein-protein interaction network was generated using the STRING database (Figure 4). The interacting proteins have important functions in the clusters of amino acid metabolism, carbohydrate/energy metabolism, photosynthesis, protein metabolism and cell processes. As shown in Figure 4, the differentially expressed proteins from different clusters connected tightly, revealing direct or indirect functional links in the network. The chloroplast heat shock protein 70-1 (cpHsc70-1) plays an important role in protein precursor import into chloroplasts and had the highest number of interactions (12) in the network. Two proteins involved in carbon fixation in photosynthetic organisms, glyceraldehyde-3-phosphate dehydrogenase B (GAPB) and phosphoglycerate kinase (AT1G56190), also had a high number of interactions (10 and 8, respectively). These proteins were connected tightly to the other enzymes involved in the glycolysis pathway, such as enolase 1 (ENO1) and pyruvate dehydrogenase E1 β subunit (T2H7.8). Moreover, these proteins with a high number of connections were present in lower amounts in the YL mutant. The interactions among these proteins may play important roles in carbohydrate metabolism and photosynthetic performance.

### 2.5. Gene Expression Analysis by qRT-PCR

**Figure 5:**
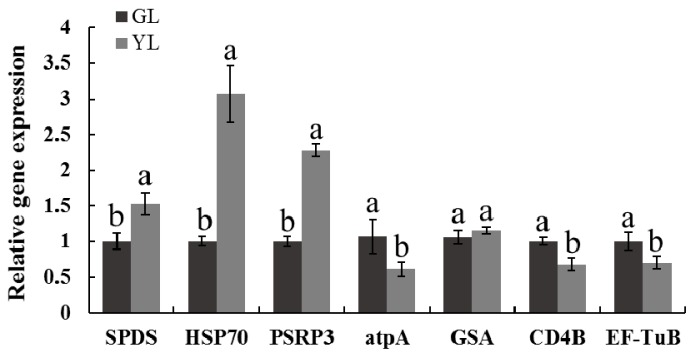
Expression levels of selected protein-encoding genes in *G. biloba*. SPDS, HSP70, PSRP3, atpA, GSA, CD4B and EF-Tu are spermidine synthase (spot 5032), heat shock 70 kDa protein (spot 5033), plastid-specific 30S ribosomal protein 3(spot 0038), ATP synthase CF1 α chain (spot 2134), glutamate-1-semialdehyde aminotransferase (spot 2239), ATP-dependent Clp protease ATP-binding subunit clpA homolog CD4B (spot 3940), and elongation factor TuB (spot 2135), respectively. Different letters indicate significant differences at *p* < 0.05.

To further verify the changes of proteins between the two colour leaves, we examined the transcript levels of selected protein-coding genes by qRT-PCR. Seven proteins involved in amino acid metabolism, cellular processes, photosynthesis, protein metabolism and chlorophyll metabolism were selected, and the expression levels of their encoded genes were analysed (Figure 5). The results showed that the expression levels of spermidine synthase (SPDS), heat shock 70 kDa protein (HSP70) and plastid-specific 30S ribosomal protein 3 (PSRP3) increased significantly in YL, and the expression levels of ATP synthase CF1 α chain (atpA), ATP-dependent Clp protease ATP-binding subunit clpA homolog CD4B (CD4B) and elongation factor TuB (EF-TuB) decreased, which is consistent with the expression patterns of their corresponding proteins. In contrast, the protein and mRNA expression patterns of glutamate-1-semialdehyde aminotransferase (GSA) differed, which indicated that some of the identified proteins are regulated at the transcriptional level and others are regulated post-transcriptionally.

## 3. Discussion

Yellow-green leaf colour mutants are common in plants and always exhibit low photosynthetic capacity and weak growth. Consequently, these mutants are considered valuable materials for photosynthesis research [13,32,33]. The photosynthesis and a wide range of other metabolisms occur in the chloroplasts, making them the largest metabolically active compartments in the mature leaf cell. Leaf colour mutants lead to impaired chloroplast development, and this damage is often considered a cause of the alteration in photosynthesis [33]. In the present study, we examined a xantha mutant of *G. biloba* and its photosynthetic and anatomic characteristics. We also performed a comparative analysis of the protein expression patterns in both leaves and chloroplasts of YL and GL as a first step towards understanding the molecular mechanisms.

### 3.1. Photosynthesis and Anatomical Characteristics

In higher plants, photosynthesis is a complex biological process in which light energy absorption and transformation occur on the thylakoid membrane of chloroplasts. The chlorophyll-protein complexes are embedded in a lipid matrix of thylakoid membranes, and its contents in chloroplasts play a regulatory role in the amount of light energy absorption, which directly determines the photosynthetic potential and primary production of plant leaves [2,34]. Chl biosynthesis is associated with the formation of thylakoid membranes and is also coordinated with chloroplast development [35,36]. The anatomical characteristics of chloroplasts have been described in several Chl-deficient mutant species, such as *O. sativa*, *A. thaliana*, *Brassica napus*, and *Triticum turgidum* [19,28,33,36,37]. In general, the chloroplasts of chlorotic mutants are not well developed, with abnormal chloroplast ultrastructures. The chloroplasts of chlorotic mutants often contain fewer, thinner and irregularly arranged grana lamellae compared with wild type, and the thylakoids usually swell at different levels. In the YL mutant, anatomical characterization revealed that this ginkgo mutant had less dense thylakoid lamellae, irregularly shaped chloroplasts, and few or no starch grains. These features are consistent with those of three previously studied rice chlorina mutants and indicate an underdeveloped chloroplast ultrastructure in the YL mutant [17,33]. These structures might lead to decreased Chl contents and abnormal Chl a/b, resulting in delayed leaf greening.

The structure of chloroplasts determines the photosynthetic capacity of plant leaves. Abnormal Chl can reduce the filling of light-harvesting complexes, resulting in low photosynthetic efficiency in Chl-deficient mutants. Most Chl-deficient mutants have lower photosynthetic efficiency than wild type, with the exception of a very few specific mutants. For example, a xantha rice mutant named *Huangyu B* has higher photosynthetic efficiency than its wild-type parent, and the photosynthetic rate of a pale-green durum wheat mutant is similar to that of wild type [37,38]. In our study, YL had lower P_n_, E and g_s_ but not C_i_ than GL, consistent with most Chl-deficient mutant plants. The Chl content in YL was approximately one-sixth of that in GL, a much more pronounced difference than those observed in other species. Leafing out and growth remained normal in the mutant because it possesses the fundamental chloroplast structure to perform photosynthesis. The fluorescence kinetics parameter ΦPSII was significantly lower in YL than in GL, indicating its low photochemical efficiency. The apparent inhibition of the photosynthetic property of the YL mutant was probably due to the low chlorophyll content and aberrant chloroplast development. Remarkably, the mutant had significantly higher NPQ than GL. NPQ reflects the nonphotochemical dissipation of the excess excitation energy, which protects the tissue from the damaging effects of reactive oxygen species (ROS) [39]. Based on these results, we suggest that PSII is inhibited in YL, resulting in increased effectiveness of the consumption and dissipation of absorbed light energy compared to GL.

### 3.2. Proteins Involved in Photosynthesis and Carbon Fixation

**Figure 6:**
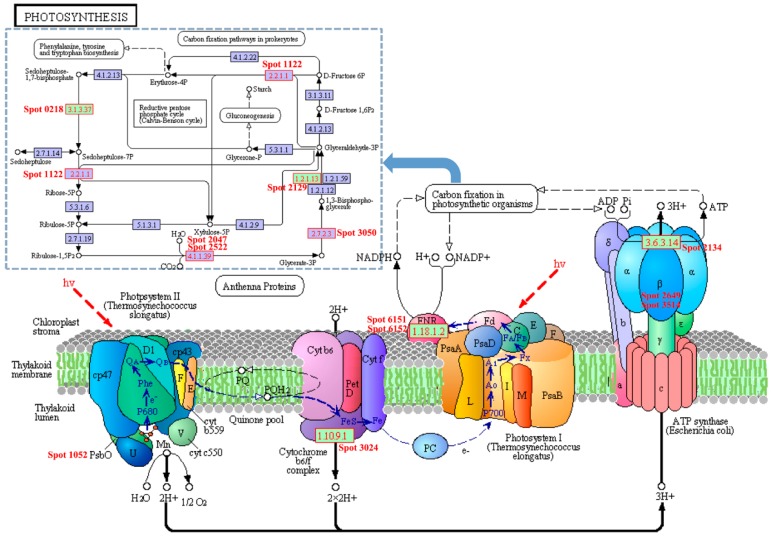
Identified proteins involved in photosynthesis in *G. biloba*. The blue dashed outline on the wireframe indicates the proteins that are involved in carbon fixation in photosynthetic organisms.

Light-dependent reactions are one set of reactions in photosynthesis and are catalysed by four major protein complexes in the thylakoid membrane: photosystem I (PSI), photosystem II (PSII), cytochrome b6f complex, and ATP synthase. These complexes work together to transform light energy into chemical forms, ATP and NADPH. In the present study, 16 differentially accumulated proteins involved in carbon fixation in photosynthetic organisms and 8 protein species involved in photosynthesis were identified in the leaves of two different colours of ginkgo. These proteins function directly in the electron transport and carbon fixation pathways (Figure 6, Table 1). Several studies have reported that some photosystem proteins in xantha mutants are deficient or present at low levels [40,41]. However, we observed that three different protein species, Rieske iron–sulphur protein precursor (PetC, spot 3024), oxygen-evolving enhancer protein 2 (OEC, spot 1052), and ferredoxin NADP reductase (FNR, spot 6151 and spot 6152), which originate from the cytochrome b6f complex PSII and PSI, respectively, were increased in YL (Figure 6). These complexes create and transfer electrons from H_2_O to NADP^+^ and ADP, executing the initial energy conversion reactions. We also detected one photosystem II stability/assembly factor HCF136 (HCF136, spot 1118) in our study, which was decreased in YL. HCF136 is an assembly factor of the photosystem II reaction centre that is localized in the lumen of stroma thylakoids [42]. Three different subunits of ATP synthase (spot 2134, atpA; spot 3514 and spot 2649, ATP synthase subunit β), however, obviously decreased. These subunits are essential to ATP synthesis and play a key role in light-reactions of photosynthesis. These fundamental metabolic alterations might be due to the low level of Chl, a component of the light-harvesting complexes. The complex changes also indicated that the thylakoids in YL were obviously affected, consistent with the chloroplast ultrastructure.

Ribulose-1,5-bisphosphate carboxylase oxygenase (Rubisco) is the initial carbon fixation enzyme of photosynthesis and catalyses the first step of the Calvin cycle for carbon assimilation [43]. Rubisco is the most abundant protein in leaves and constitutes nearly 50% of the soluble leaf protein in C3 plants. There are multiple forms of this protein in plant leaves, and there is a dynamic balance between Rubisco and its degraded forms, complicating the determination of the abundance of Rubisco by 2-DE [44]. In this study, four spots were identified as Rubisco large subunits (RLS), two of which (spot 1024 and spot 2216) were increased and the others (spot 2522 and spot 2047) were decreased. Rubisco activase is a catalytic chaperone for Rubisco; it changes the conformation of inactive Rubisco to the active form using the energy from ATP hydrolysis [45]. In this study, three types of Rubisco activase (RCA, spot 0223, spot 0021, spot 5144) were markedly increased in YL, indicating that the activation of Rubisco was increased. We also observed that the other four protein species participating in the Calvin cycle declined in abundance, including sedoheptulose-1,7-bisphosphatase precursor (SBP, spot 0218), glyceraldehyde-3-phosphate dehydrogenase B (GAPB, spot 2129), and phosphoglycerate kinase (PGK, spot 3050). These changes indicate that YL has a low CO_2_ fixation capacity, which slows the photosynthetic process.

### 3.3. Proteins Involved in Cellular Processes

As a principal component of microfilaments of cytoskeletons in eukaryotic cells, actin plays an important role in cell development, movement, differentiation and cellular components. Actin filaments associated with the surface of chloroplasts are supposed to function as an anchor for the chloroplast and to maintain cell structure; therefore, actin can be used as marker of cytoplasmic protein when examining the purity of isolated chloroplasts [25,46]. The ATP-dependent zinc metalloprotease FtsH family is a large, well-characterized family of chloroplast proteases that is involved in the degradation of unassembled proteins and the turnover of photosynthetic protein complexes in thylakoid membranes [20,47]. FtsH proteins are also suggested to play a direct or indirect role in chloroplast biogenesis, photosystem II repair, and complex formation [48,49]. The disruption of either FtsH2 or FtsH5 results in some degree of leaf variegation, and the double mutant of FtsH2 and FtsH8 exhibits an albino-like phenotype in *Arabidopsis*, indicating that the subunits are necessary for the pool of FtsH isomers that are important for chloroplast biogenesis [20,49]. A previous study also demonstrated that the levels of FtsHs, including FtsH2, FtsH8, and FtsH5, were significantly reduced in a leaf variegation thf1 mutant compared with wild type but were increased in a virescent ClpR4-3 thf1 mutation [50]. ClpA is an ATP-dependent chaperone of caseinolytic proteases (Clp) that mainly exists in chloroplasts and is essential for the biogenesis and function of chloroplasts [51]. In this study, spot 4031, spot 2240 and spot 3055 were identified as FtsH proteases, spot 3940 and spot 3939 were identified as CD4B, and all were reduced in YL, which might be attributable to the underdeveloped chloroplasts. Additionally, the decrease in CD4B in the mutant might decrease the formation and maintenance of functional thylakoid electron transport [52].

ROS are produced by aerobic metabolic processes in chloroplasts, mitochondria, and peroxisomes, such as respiration and photosynthesis. ROS can cause oxidative damage to the membrane system but simultaneously also plays many signalling roles in plants in regulating development and mediating stress responses [53]. The scavenging of ROS in cells is regulated by antioxidants such as ascorbate peroxidase (APX), superoxide dismutase (SOD), and catalase (CAT). In the present study, three enzymatic antioxidants, ascorbate peroxidase (APx, spot 1051 and spot 0040), FeSOD (spot 0041) and copper–zinc superoxide dismutase (Cu/ZnSOD, spot 5015), involved in redox homeostasis were identified as increased. As members of the defence system against ROS in chloroplasts, APx and SOD are the key enzymes catalysing the conversion of H_2_O_2_ to H_2_O. FeSOD and Cu/ZnSOD are a different class of SODs that is identified by their metal co-factor, and both are considered ROS-scavenging enzymes [54,55]. The YL mutant may be more sensitive to photo-induced damage and ROS accumulation and therefore had a higher NPQ and an increased abundance of APx and SOD in chloroplasts to detoxify the ROS damage and maintain cellular homeostasis. However, two peroxiredoxins (Prx, spot 1028 and spot 4015) decreased in the YL mutant. In YL, most Prx might be chloroplastic, whereas in GL, most Prx might be cytosolic.

### 3.4. Proteins Involved in Carbohydrate Metabolism

In addition to the 24 specific proteins discussed in Section 3.2 related to the photosynthetic pathway in chloroplasts, 12 proteins associated with carbohydrate metabolism were identified in this study. Four enzymes (spot 5031, spot 2137, spot 7023, spot 1025) were involved in the glycolysis pathway, three of which (spot 2137, spot 7023, spot 1025) exhibited a decreased expression pattern in YL. The pyruvate dehydrogenase multienzyme complex (PDH) plays a key role in carbohydrate metabolism and catalyses the oxidative decarboxylation of pyruvate to generate acetyl-CoA. As a subunit of PDH, pyruvate dehydrogenase E1 (PDH E1) catalyses the oxidative decarboxylation of pyruvate and transfers the hydroxyethyl group to thiamine diphosphate (ThDP) [56,57]. PDH E1 (spot 7023) was less abundant in YL, which might suggest that the mutant maintained a low level of acetyl-CoA. Myo-inositol-1-phosphate synthase (MIPS, spot 2133) also declined in abundance. MIPS catalyses the conversion of d-glucose 6-phosphate to 1-l-myo-inositol-1-phosphate (MIP), providing the immediate precursor to myo-inositol, which is an intermediate carbon skeleton of cell wall uronic acids and pentoses derived from d-glucose [58]. MIPS has been found to play a key role in the biosynthesis of inositol and inositol phosphate and to be important in plant development and environmental stress tolerance [59].

In chloroplasts, starch is the major energy storage compound in the form of granules and is used as a primary store of excess carbohydrate produced during photosynthesis. We identified four protein species associated with starch metabolism that differed in abundance, including one protein that increased, ADP-glucose pyrophosphorylase small subunit (AGPase, spot 4022), and three proteins that decreased: starch synthase (SS, spot 3051) and granule-bound starch synthase (GBSS, spot 3049 and spot 3841). They play a major role in the regulation of starch synthesis. AGPase catalyses the conversion of glucose-1-phosphate to ADP-glucose, the substrate for starch synthesis [60]. GBSS is a type of SS and is a key enzyme in the formation of amylose found in starches [61]. The decreased abundance of SS and GBSS might suggest that energy is stored at low levels in YL. This conclusion is supported by the chloroplast ultrastructure: starch grain accumulation was rare in YL compared with GL, as shown in Figure 1C,D. The decrease in these differentially accumulated proteins might suggest that carbohydrate metabolism is reduced in YL.

### 3.5. Proteins Involved in Protein Metabolism

The biosynthesis, metabolism and transport of proteins in chloroplasts are crucial for chloroplast biogenesis and development [62]. A total of 14 identified proteins in our study are involved in protein metabolism. Three protein species related to protein biosynthesis, initiation factor eIF5-A (eIF5-A, spot 2042), translation elongation factor EF-G (EF-G, spot 1119) and EF-TuB (spot 2135), were decreased in the YL mutant. The protein eIF5-A is thought to play an important role in the translation machinery; it stimulates methionyl-puromycin synthesis for the formation of the first peptide bond [63]. EF-G and EF-Tu catalyse the elongation cycle of translation, which functions in the sequential addition of amino acids to the growing polypeptide chain [64]. A *snowy cotyledon 1* mutant of *Arabidopsis* containing a point mutation of the *EF-G* gene exhibits white cotyledons during the early development stage [65]. The protein species eIF5-A, EF-G and EF-Tu work together to perform initiation and elongation of the newly growing peptide chains. The decreased abundance of these proteins in YL might inhibit protein biosynthesis in the chloroplasts. Two heat shock protein 60 proteins (Hsp60, spot 2628 and spot 0641) and six heat shock 70 kDa proteins (Hsp70, spot 6020, spot 5033, spot 3719, spot 0846, spot 2848 and spot 2045), which belong to the chaperone family and are involved in protein folding and assembly, were differentially expressed. Hsp60 was originally identified as the Rubisco ligase protein and may combine the large and small subunits of newly synthesized Rubisco to prevent incorrect aggregation. Hsp60 is also supposed to assist with the folding and assembly of proteins within chloroplasts [66]. Hsp70 is a type of molecular chaperone that has the essential function of assisting with protein folding processes, preventing protein aggregation, and targeting unstable proteins for proteolytic degradation. These chaperones appear to play specific roles according their subcellular location. For instance, cytosolic Hsp70 assists protein folding and prevents aggregation, and chloroplastic Hsp70 is involved in the import and translocation of a precursor protein [67]. We observed four Hsp70s that increased significantly and two chloroplast HSP70s that decreased in the YL, which might imply that protein folding and assembly in were enhanced in YL, whereas translation decreased. Additionally, proteasome subunit β (spot 3023) increased in YL, which may indicate enhanced protein proteolysis in the mutant. Based on these findings, the identified proteins involved in protein metabolism were largely implicated in the inhibition of protein biosynthesis and the enhancement of protein folding and assembly in the YL mutant.

### 3.6. Proteins Involved in Other Metabolic Pathways

Glutamine synthetase (GS) is a key enzyme in the plant primary nitrogen assimilatory process because it catalyses the conversion of ammonium to form glutamine and other related nitrogenous compounds, which are important substrates for protein synthesis. GS has different subcellular localizations, and the major role of chloroplastic GS is thought to be the reassimilation of ammonia generated in photorespiration [68]. In this study, two chloroplastic GS (spot 2048 and spot 3048) decreased significantly, implying that N assimilation was inhibited in the chloroplasts of YL. Cysteine synthase (CSase) catalyses the synthesis of cysteine from *O*-acetylserine and disulphides. Cysteine is the only amino acid that contains disulphide bonds (S–S) and protects cellular environments from oxidative stress. The formation of S–S is a key step in the activation of molecular chaperones activation and is an essential substance for the correct folding and stability of some proteins [69]. Spermidine synthase (SPDS) is a key enzyme that participates in polyamine biosynthesis in plants and plays a pivotal role in plant defence against environmental stresses as a signalling regulator [70]. Here, we identified two protein species, CSase (spot 0129) and SPDS (spot 5032), that were more abundant in YL than in GL, which might be related to the increase in molecular chaperone activity and the decrease in the effect of oxidative stress.

GDP-d-mannose-3′,5′-epimerase (GME) catalyses the conversion of GDP-l-galactose from GDP-d-mannose, which precedes the critical step of the ascorbate biosynthesis pathway. GME also plays an important role at the intersection between ascorbate and non-cellulosic cell-wall polysaccharide biosynthesis in higher plants [71]. Ascorbate is another major antioxidant in plants that prevents cells from ROS damage generated by cellular processes as well as in vitro stress. Spot 1048 was identified as GME and exhibited higher abundance in the YL mutant. Glutamate-1-semialdehyde 2,1-aminomutase (GSA) is a key enzyme in plant tetrapyrrole biosynthesis that catalyses the synthesis of δ-aminolevulinic acid, a precursor in chlorophyll and heme biosynthesis [72]. In the present experiment, GSA (spot 2239) decreased significantly in YL, which might suggest the likely reason for the xanthas phenotype was the reduction of a precursor in the chlorophyll biosynthesis pathway. By contrast, there were no significant differences in the mRNA abundance of GSA between these two types of leaves, which might suggest that this identified protein is regulated at the post-transcriptional level.

In addition, our results indicated that the 2-DE profiles of total leaf proteins and chloroplast proteins presented similar protein patterns, which was in accordance with that found in *Glycine max* and *Kandelia candel* [73,74]. It is most likely because chloroplasts in leaf cell are numerous and are rich in soluble proteins, such as Rubisco, Rubisco activase, and ATP synthase, which account for more than half of the total soluble proteins in mature leaf cells [43,75,76]. The bioinformatic analysis with WolfPsort revealed that nearly 82% of the identified proteins from chloroplast were predicted to be typical chloroplast proteins, indicating the high purity of chloroplast isolation. Since proteins were searched against the viridiplantae database of NCBI, many differential proteins such as Rubisco large subunit were identified as orthologous complexes in other organisms [77,78]. Our results also revealed that 82 of the 89 identified proteins appeared to be 60 unique kinds of proteins, suggesting these sets of proteins might be isoforms due to the post-translational modifications or belong to the same protein families. These findings provided evidence on how the isoforms or family proteins in chloroplast modulate the complex metabolic network in YL.

## 4. Materials and Methods 

### 4.1. Plant Materials

Similar sized leaves of two ginkgo leaf types (YL and GL) with uniform genetic backgrounds were collected from the 150-year old mutant tree in April. The sample leaves were stored in the dark at 4 °C for 24 h until chloroplast isolation. Leaves for protein and RNA extraction were collected from one-year-old lateral branches on the sun-exposed side, immediately frozen in liquid nitrogen and stored in a −70 °C freezer until use. Each sample consisted of 20 leaves.

### 4.2. Pigment Determination and Photosynthetic Characteristics

To investigate the changes in the photosynthetic physiology, we analysed the chlorophyll content, gas-exchange parameters and chlorophyll fluorescence in the two leaves of different colours. Chlorophyll was extracted and quantified using a spectrophotometer as previously described by Tang et al. [79]. Green and yellow leaves (0.2 g fresh weight) were prepared and extracted with a mixed extraction solution (acetone:alcohol:distilled water = 4.5:4.5:1, volume ratio) until complete bleaching. The concentrations of chlorophyll were calculated based on the absorbance of the solution at 645 and 663 nm. The chlorophyll content (chlorophyll a and b) was measured with six replicates.

The net photosynthesis rate (P_n_), transportation rate (E), stomatal conductance (g_s_) and internal CO_2_ concentration (C_i_) were measured with a CIRAS-2 portable open system gas analyser (PP Systems, Haverhill, MA, USA) at an ambient temperature of approximately 25 °C, a CO_2_ concentration of 400 µmol∙mol^−1^, relative humidity of 60% and photosynthetic photon flux density (PPFD) of 1500 µmol∙m^−2^∙s^−1^. The chlorophyll fluorescence was measured with a FMS-2 pulse modulated fluorometer (Hansatech Instruments, King’s Lynn, UK) as described by Xu et al. [80]. To measure the maximum quantum yield of photosystem II (Fv/Fm), leaves were dark-adapted for at least 30 min. The effective quantum yield of photosystem II (PSII) electron transport (ΦPSII = (Fm′ − Fs)/Fm′), the efficiency of excitation energy capture by open PSII centres (Fv′/Fm′), nonphotochemical quenching (NPQ = (Fm − Fm′)/Fm′), photochemical quenching (q_p_ = (Fm′ − Fs)/(Fm′ − Fo′) were calculated. The gas exchange parameters and chlorophyll fluorescence were recorded between 09:00 and 11:00 on sun-exposed leaves of similar sizes on one-year-old lateral branches.

### 4.3. Transmission Electron Microscopy Analysis

To observe the ultrastructure of chloroplasts, samples of GL and YL were fixed in 2.5% glutaraldehyde solution in 100 mM phosphate buffer (pH 7.2) for 24 h at 4 °C and postfixed with 1% osmium tetroxide (OsO_4_) in deionized water. The postfixed tissues were rinsed three times with phosphate buffer, followed by dehydration in a graded ethanol series (30%, 50%, 70%, 80%, 90%, 100%, 100%) of 20 min each. After embedding in an Epoxy resin composed of a mixed solution of Epon812, the samples were sectioned with a glass knife using an LKB-V ultramicrotome (LKB, Bromma, Sweden). Ultra-thin sections were deposited on copper grids and stained with uranyl acetate and lead citrate. TEM experiments were conducted on a JEM-2100 transmission electron microscope (JEOL Ltd., Tokyo, Japan).

### 4.4. Isolation of Chloroplasts

Chloroplasts were isolated according to Diekmann et al. [81], with minor modifications. Leaves (100 g fresh weight) were homogenized in 600 mL of an ice-cold buffer (1.25 M NaCl, 50 mM Tris-HCl pH 8.0, 7 mM EDTA, 5% PVP-40, 0.1% BSA, 1 mM DTT) using a commercial blender, and all subsequent operations were performed at 4 °C. Chloroplasts were purified using 30% sucrose solution (30% sucrose in 50 mM Tris-HCl pH 8.0, 25 mM EDTA) and dissolved in wash buffer (0.35 M sorbitol, 50 mM Tris-HCl pH 8.0, 25 mM EDTA). The chloroplast solution was layered carefully on top of pre-chilled sucrose gradients (prepared using 30% and 52% sucrose solution) and centrifuged at 14,600× *g* for 70 min (4 °C). Intact chloroplasts were collected from the interphase of the 52% to 30% layers using a wide-bore pipette, diluted with wash buffer, and then directly used in protein extraction or stored at −80 °C. The purified chloroplasts were visualized under a DM5000B fluorescence microscope (Leica, Wetzlar, Germany).

### 4.5. Protein Extraction and Quantification

The procedure for extracting proteins from chloroplasts was modified from the improved phenol (BPP) method [82]. Chloroplast pellets were re-suspended uniformly in ice-cold extraction buffer containing 100 mM Tris (pH 8.0), 100 mM EDTA, 50 mM borax, 50 mM vitamin C, 1% Triton X-100, 2% β-mercaptoethanol, and 30% sucrose. Two volumes of Tris-saturated phenol (pH 8.0) were added to the solution, followed by vortexing for 10 min at room temperature. After the sample was centrifuged at 15,000× *g* for 15 min (4 °C), the upper phase was transferred to a new centrifuge tube. The chloroplast proteins were centrifuged at 15,000× *g* for 15 min (4 °C) and precipitated by adding ammonium sulphate-saturated methanol. The protein pellets were washed twice with ice-cold acetone and then lyophilized on an ALPHA 2-4 LD plus freezer dryer (Christ, Osterode, Germany).

Total proteins were extracted from leaves according to the acetone/trichloroacetic acid (TCA) precipitation method [83]. The leaf sample powder was suspended in acetone buffer containing 10% TCA and 0.07% β-mercaptoethanol and incubated at −20 °C for 2 h. After centrifugation at 15,000× *g* for 20 min (4 °C), the resultant precipitate was washed three times with ice-cold acetone and then lyophilized. The obtained protein powder was dissolved in lysis buffer containing 7 M urea, 2 M thiourea, 4% CHAPS, 1% dithiothreitol (DTT), 0.5% IPG buffer pH 3–10. The protein concentration was quantified according to the Bradford method [84].

### 4.6. 2-D Electrophoresis and Gel Imaging Analysis

Two-dimensional electrophoresis was performed using a GE Healthcare 2-DE system (GE Healthcare, Piscataway, NJ, USA) according to the manufacturer’s instructions, with some modifications. Approximately 350 μg of protein were loaded onto a 24 cm nonlinear gradient IPG strip (pH 4–7 and 3–10), and the strips were loaded into an Ettan IPGpho 3 IEF System (GE). The separation of proteins in the second dimension was performed on an SDS-PAGE gel (12.5% polyacrylamide) using the Ettan Daltsix apparatus (GE). The signal was visualized by silver staining. Three biological replicates were prepared for each type of leaf.

The gel images were digitized with an Image scanner III (GE), and analysed with the PDQuest software package (Version 8.0.1, Bio-Rad, New York, NY, USA). Spots were detected, matched, and normalized on the basis of the total density of gels with the parameter of percentage volume according to the manufacturer’s instructions. For each protein spot, the average spot quantity value and its variance coefficient at each sample were determined. One-way analysis of variance (ANOVA) was performed at *p* < 0.05 to assess for absolute protein changes between the two leaves of different colours. Spots with a mean abundance that changed more than 1.5-fold or less than 0.66-fold in different leaves were considered differentially accumulated proteins and selected for protein identification.

### 4.7. Protein Digestion and MALDI-TOF-TOF Analysis

Protein spots were rehydrated in digestion solution with sequencing grade modified trypsin as described previously [85]. After digestion, the tryptic peptides were extracted, lyophilized and stored at −80 °C. All peptide mass fingerprint data were obtained using an UltrafleXtreme MALDI TOF/TOF mass spectrometer (Bruker Daltonics, Bremen, Germany) with flexAnalysis software. All spectra of proteins were submitted to database searching using the MASCOT search program (available online: http://www.matrixscience.com), against NCBI database (Viridiplantae). The searching parameters were as follows: 100 ppm mass accuracy, trypsin cleavage one missed cleavage allowed, carbamidomethylation of Cys as fixed modification, oxidation of Met was allowed as variable modification, and MS/MS fragment tolerance was set to 0.6 Da. The subcellular locations of the differentially accumulated proteins in this study were predicted using the publicly available program, WolfPsort (available online: http://www.genscript.com/tools/wolf-psort/) [86]. Differentially accumulated proteins were functionally classified based on Kyoto Encyclopedia of Genes and Genomes (KEGG) pathway analysis (available online: http://www.kegg.jp/kegg/pathway.html) and the literature, and the protein–protein interaction network was evaluated using STRING 10 (STRING is a database of known and predicted protein-protein interactions, which is available online: http://string-db.org) against the *A. thaliana* database.

### 4.8. RNA Extraction and qRT-PCR Analysis

Total RNA was extracted from leaves using the MiniBEST Plant RNA Extraction Kit (Takara, Otsu, Japan) according to the manufacturer’s instructions. The quality of the RNA was determined by agarose gel electrophoresis, and the concentration was detected by UV spectrophotometric analysis. The reverse transcription-polymerase chain reaction was performed using a Transcriptor First Stand cDNA Synthesis Kit (Roche, Mannheim, Germany). Real-time quantitative PCR (qRT-PCR) was performed on ABI PRISM 7500 Sequence Detection System (Applied Biosystems, Foster City, CA, USA) using SYBR Green PCR Master Mix (Roche). The glyceraldehyde-3-phosphate dehydrogenase gene (*GbGAPDH*, GenBank Accession no. L26924) was used as the reference gene [87]. The gene-specific primers for *GbGAPDH* and selected protein-coding genes were designed based on the transcriptome sequencing performed previously (Table 2). The qRT-PCR data were calibrated using the 2^−ΔΔ*C*t^ method for relative quantification between different colours of leaves [88]. Three biological replicates were prepared for each sample.

### 4.9. Statistical Analysis

Parametric data are presented as means ± SD. Statistical analyses were performed by one-way analysis of variance (ANOVA), and mean differences were compared by the lowest standard deviations (L.S.D.) test using SPSS 17.0.

## 5. Conclusions 

In the present study, the morphological, photosynthetic and proteomic characteristics of YL and GL variations of ginkgo were analysed. The YL mutant had a relatively low chlorophyll content, photosynthetic properties and undeveloped chloroplasts, with few or no starch grains. Proteomic analysis of both leaves and chloroplasts of the two different colours of leaves was performed using 2-DE technology coupled with MALDI-TOF/TOF MS. A total of 89 proteins were successfully identified, and 82 of these proteins were assigned clear functions belonging to nine metabolic pathways and processes. Notably, these identified proteins were largely enriched in protein and carbohydrate metabolism, including photosynthesis, carbon fixation in photosynthetic organisms, carbohydrate/energy metabolism, amino acid metabolism, and protein metabolism, suggesting the ginkgo mutant has specific differences in regulating cellular processes. The proteomic comparison of the two leaves of different colours revealed that the YL mutant had a severe effect on the photosynthetic machinery, with a reduction of CO_2_ fixation efficiency and reduced ATP synthesis, and thus caused a decrease in primary carbon metabolism. The identification of proteins involved in protein metabolism indicated that the mutant has an inhibitory effect on protein biosynthesis and enhanced protein folding and assembly. Furthermore, proteomic analysis identified several antioxidant proteins that are involved in redox homeostasis and might improve the defensive ability of cells against ROS damage. The identification of these differentially accumulated proteins reveals the presence of a complex metabolic network in YL and the possible reason for the variation in leaf colour. Future studies should examine more leaf developmental stages to elucidate the regulation of pigment biosynthesis or the changes in the colour pattern and to evaluate the alteration of comprehensive metabolic networks of this leaf colour mutant.

## Figures and Tables

**Table 1:** Identification and database search of differentially accumulated proteins in *G. biloba*.

Spot No.^a^	Accession No. (GI) ^b^	Protein Name ^c^	Species ^c^	Exp kD/pI ^d^	Theor kD/pI ^e^	Score ^f^	SC ^g^	MP ^h^	FC ^i^	E ^j^	Sub-CL ^k^
**Amino acid metabolism**											
9708	gi|356508448	5-methyltetrahydropteroyltriglutamate-homocysteine methyltransferase-like	*Glycine max*	66.2/6.88	89.1/6.41	59	4%	2	1.80	T	Chlo
4026	gi|563247	Acetolactate synthase precursor	*Xanthium* sp.	33.9/5.64	70.8/7.03	91	5%	2	0.33	C	Chlo
0129	gi|2493895	Cysteine synthase	*Citrullus lanatus*	35.1/4.78	34.5/6.25	79	7%	2	2.06	T	Cyto
4115	gi|99698	Glutamate-ammonia ligase	*Arabidopsis thaliana*	36.8/5.62	40.9/5.40	225	8%	2	1.68	T	Cyto
2048	gi|225432496	Glutamine synthetase leaf isozyme, chloroplastic	*Vitis vinifera*	34.1/5.26	48.3/7.57	114	4%	1	0.18	C	Chlo
3048	gi|121334	Glutamine synthetase PR-1	*Phaseolus vulgaris*	25.1/5.44	39.3/5.78	117	4%	2	0.28	C	Cyto
1049	gi|357144704	Glycerate dehydrogenase-like	*Brachypodium distachyon*	19.9/4.61	42.2/6.68	64	5%	1	5.37	C	Chlo
2046	gi|288063	Ketol-acid reductoisomerase	*Arabidopsis thaliana*	33.6/5.29	64.3/6.55	112	5%	2	0.18	C	Chlo
1054	gi|1709006	*S*-adenosylmethionine synthase 3	*Actinidia chinensis*	31.3/5.13	39.8/6.20	185	13%	3	0.19	C	Cyto
2116	gi|356505256	*S*-adenosylmethionine synthase 3-like isoform 1	*Glycine max*	34.8/5.21	43.2/6.13	312	14%	4	1.53	T	Cyto
5032	gi|2821961	Spermidine synthase	*Arabidopsis thaliana*	33.3/5.81	32.7/4.97	82	11%	3	2.03	C	Chlo
**Biosynthesis of secondary metabolites**											
2132	gi|53830379	Anthocyanidin reductase	*Ginkgo biloba*	37.8/5.02	37.6/5.63	89	4%	2	0.17	C	Chlo
2218	gi|356566889	LOW QUALITY PROTEIN: (+)-neomenthol dehydrogenase-like	*Glycine max*	39.1/5.15	57.9/7.49	55	2%	2	1.51	T	Cyto
1048	gi|225380888	GDP-d-mannose-3′,5′-epimerase	*Malus domestica*	21.2/4.71	42.9/6.25	79	8%	2	2.31	C	Cyto
2239	gi|1170029	Glutamate-1-semialdehyde 2,1-aminomutase, chloroplastic	*Hordeum vulgare*	31.1/5.07	49.7/6.39	148	11%	4	0.10	C	Chlo
**Carbohydrate/Energy metabolism**											
5031	gi|344190186	Enolase	*Corylus heterophylla*	20.4/5.88	49.4/5.62	119	7%	2	12.43	C	Chlo
2137	gi|15221107	Enolase 1	*Arabidopsis thaliana*	36.2/5.15	51.8/5.79	119	6%	2	0.61	C	Chlo
7023	gi|2982328	Pyruvate dehydrogenase E1 β subunit	*Picea mariana*	32.4/6.49	32.0/5.73	66	5%	2	0.32	C	Chlo
4022	gi|111660950	ADP-glucose pyrophosphorylase small subunit	*Citrus sinensis*	19.2/5.62	57.3/6.73	171	7%	3	2.31	C	Chlo
3841	gi|228210	Granule-bound starch synthase	*Solanum tuberosum*	80.9/5.32	67.1/6.92	138	2%	1	0.32	C	Chlo
3049	gi|15223331	Granule-bound starch synthase 1	*Arabidopsis thaliana*	24.4/5.34	67.5/8.76	175	5%	2	0.31	C	Chlo
0740	gi|17939849	Mitochondrial F1 ATP synthase β subunit	*Arabidopsis thaliana*	66.3/4.82	63.6/6.52	308	13%	5	1.80	C	Cyto
2133	gi|351767989	Myo-inositol-1-phosphate synthase	*Triticum aestivum*	39.1/5.08	56.3/5.64	67	4%	2	0.30	C	Cyto
1025	gi|115440691	Os01g0817700	*Oryza sativa* Japonica Group	35.2/4.66	61.0/5.42	134	4%	3	0.62	T	Cyto
4029	gi|356567630	2-methylene-furan-3-one reductase	*Glycine max*	34.1/5.64	42.0/8.96	337	10%	4	0.11	C	Chlo
3056	gi|255544584	phosphoglycerate kinase, chloroplastic	*Glycine max*	35.1/5.23	50.1/8.74	293	13%	4	0.12	C	Chlo
3051	gi|1172159	Starch synthase	*Ipomoea batatas*	21.8/5.48	67.9/7.47	171	13%	5	0.26	C	Chlo
**Carbon fixation in photosynthetic organisms**											
0218	gi|118175929	Chloroplast sedoheptulose-1,7-bisphosphatase	*Morus alba var.* multicaulis	43.2/4.73	42.8/6.06	92	7%	2	0.63	C	Chlo
2129	gi|120664	Glyceraldehyde-3-phosphate dehydrogenase B, chloroplastic	*Spinacia oleracea*	35.4/5.11	48.6/6.72	140	6%	2	0.58	C	Chlo
3050	gi|15223484	Phosphoglycerate kinase	*Arabidopsis thaliana*	23.7/5.32	50.0/8.27	398	12%	5	0.62	C	Chlo
2522	gi|132000	Ribulose bisphosphate carboxylase large chain	*Nicotiana acuminata*	53.2/5.09	53.4/6.41	322	6%	3	0.64	T	Cyto
3047	gi|1006698	Rubisco subunit binding protein, β subunit	*Pseudotsuga menziesii*	19.2/5.31	9.7/4.37	151	18%	1	9.31	C	Chlo
1024	gi|161777955	Ribulose-1,5-bisphosphate carboxylase/oxygenase large subunit	*Luculia pinceana*	42.8/4.89	52.2/6.17	171	7%	2	3.16	T	Chlo
2216	gi|161777955	Ribulose-1,5-bisphosphate carboxylase/oxygenase large subunit	*Luculia pinceana*	40.8/5.18	52.2/6.17	238	7%	3	2.10	T	Chlo
2047	gi|12098	Ribulose-1,5-bisphosphate carboxylase/oxygenase large subunit	*Afrocarpus falcatus*	34.9/5.29	53.1/5.91	121	3%	2	0.21	C	Chlo
0223	gi|337263422	Chloroplast rubisco activase	*Ophiopogon japonicus*	40.5/4.46	48.0/6.04	87	3%	1	5.05	C	Chlo
0021	gi|10720249	Rubisco activase	*Vigna radiata var.* radiata	33.4/4.51	48.0/7.57	177	13%	4	1.79	T	Chlo
5144	gi|4261547	Rubisco activase	*Spinacia oleracea*	40.6/6.06	47.8/7.67	141	3%	1	2.08	C	Chlo
2627	gi|170129	Rubisco activase precursor	*Spinacia oleracea*	59.1/5.03	51.7/6.28	171	5%	2	0.61	T	Chlo
2646	gi|170129	Rubisco activase precursor	*Spinacia oleracea*	55.2/5.22	51.7/6.28	93	3%	1	0.20	C	Chlo
2651	gi|170129	Rubisco activase precursor	*Spinacia oleracea*	55.2/5.32	51.7/6.28	167	5%	2	0.25	C	Chlo
0561	gi|170129	Rubisco activase precursor	*Spinacia oleracea*	52.8/4.86	51.7/6.28	160	5%	2	7.04	C	Chlo
1122	gi|255541252	Transketolase, putative	*Ricinus communis*	38.8/5.01	81.6/6.52	199	8%	5	1.55	T	Chlo
**Cellular process**											
2339	gi|20329	Actin	*Oryza sativa* Indica Group	44.2/5.09	42.2/5.72	81	4%	1	0.48	C	Cyto
8221	gi|227069391	Actin 4	*Picea abies*	41.2/6.59	41.9/5.31	298	16%	4	2.29	T	Cyto
3940	gi|399213	ATP-dependent Clp protease ATP-binding subunit clpA homolog CD4B, chloroplastic	*Solanum lycopersicum*	110.2/5.35	102.4/5.86	207	4%	3	0.29	C	Chlo
3939	gi|399213	ATP-dependent Clp protease ATP-binding subunit clpA homolog CD4B, chloroplastic	*Solanum lycopersicum*	110.2/5.34	102.4/5.86	230	8%	5	0.41	C	Chlo
4031	gi|42561751	ATP-dependent zinc metalloprotease FtsH 8	*Arabidopsis thaliana*	24.8/5.51	73.3/5.72	202	11%	6	0.33	C	Chlo
2240	gi|2492515	ATP-dependent zinc metalloprotease FtsH, chloroplastic	*Capsicum annuum*	30.2/5.29	71.2/6.55	131	11%	4	0.41	C	Chlo
2130	gi|728744	Auxin-induced protein PCNT115	*Nicotiana tabacum*	35.2/5.03	34.3/7.10	120	9%	3	0.35	C	Nucl
3055	gi|6692685	F12K11.22	*Arabidopsis thaliana*	30.2/5.42	71.0/5.81	248	12%	6	0.16	C	Chlo
**Lipid metabolism**											
3026	gi|210110834	β-hydroxyacyl-ACP dehydrase 1	*Arachis hypogaea*	24.3/5.31	24.1/9.10	112	12%	3	0.27	T	Chlo
0137	gi|29367475	Fibrillin-like protein	*Oryza sativa* Japonica Group	35.3/4.65	33.9/5.04	126	6%	3	2.02	T	Chlo
0322	gi|339697596	Stearoyl-ACP desaturase	*Ginkgo biloba*	44.6/4.55	47.2/6.14	157	14%	4	1.83	C	Chlo
**Photosynthesis**											
2134	gi|380356155	ATP synthase CF1 α chain (chloroplast)	*Ginkgo biloba*	38.2/5.13	55.8/5.02	440	18%	8	0.25	C	Chlo
3514	gi|3913118	ATP synthase subunit β, chloroplastic	*Picea abies*	53.3/5.12	52.6/5.18	224	10%	3	0.65	T	Chlo
2649	gi|3913118	ATP synthase subunit β, chloroplastic	*Picea abies*	55.3/5.29	52.6/5.18	523	20%	6	0.39	C	Chlo
6151	gi|119904	Nicotinamide adenine dinucleotide phosphate (NADP) reductase, leaf isozyme, chloroplastic	*Pisum sativum*	37.4/6.09	40.5/8.56	119	6%	1	4.41	C	Chlo
6152	gi|119904	NADP reductase, leaf isozyme, chloroplastic	*Pisum sativum*	37.5/6.11	40.5/8.56	146	6%	1	8.67	C	Chlo
1052	gi|131390	Oxygen-evolving enhancer protein 2, chloroplastic	*Pisum sativum*	29.8/4.81	28.2/8.29	158	18%	3	1.79	C	Chlo
1118	gi|225423755	Photosystem II stability/assembly factor HCF136, chloroplastic	*Vitis vinifera*	36.1/5.09	44.5/6.92	370	19%	6	0.50	C	Chlo
3024	gi|351726724	Rieske iron-sulphur protein precursor	*Glycine max*	24.8/5.23	24.5/9.01	95	18%	3	2.37	T	Chlo
**Protein metabolism**											
0641	gi|15222729	Chaperonin 60 subunit β 1	*Arabidopsis thaliana*	57.9/4.79	64.2/6.21	57	2%	1	1.94	C	Chlo
6020	gi|123601	Heat shock 70 kDa protein	*Glycine max*	31.3/5.71	71.3/5.37	415	10%	6	2.49	T	Cyto
5033	gi|585273	Heat shock 70 kDa protein	*Solanum tuberosum*	33.4/6.02	73.3/6.37	190	3%	2	7.63	C	Mito
3719	gi|357112870	Heat shock cognate 70 kDa protein 2-like	*Brachypodium distachyon*	66.7/5.45	71.6/5.09	259	10%	5	1.51	T	Cyto
0846	gi|357112870	Heat shock cognate 70 kDa protein 2-like	*Brachypodium distachyon*	70.2/4.49	71.6/5.09	355	11%	6	2.26	C	Cyto
2628	gi|24637539	Heat shock protein 60	*Prunus dulcis*	58.8/5.04	58.1/5.26	102	5%	2	0.66	T	Cyto
2848	gi|166919370	Chloroplast heat shock protein 70-1	*Ipomoea nil*	81.3/5.18	74.5/5.14	497	9%	5	0.48	C	Chlo
2045	gi|124245039	Chloroplast heat shock protein 70 (HSP70)	*Cucumis sativus*	35.1/5.26	75.5/5.18	721	16%	10	0.34	C	Chlo
0039	gi|242076604	Hypothetical protein SORBIDRAFT_06g023840	*Sorghum bicolour*	19.3/4.55	85.3/5.42	151	8%	4	7.75	C	Chlo
3023	gi|224084924	Proteasome subunit β type 3-2 family protein	*Populus trichocarpa*	27.2/5.23	23.1/5.18	215	17%	3	2.41	T	Cyto
0038	gi|255576477	Plastid-specific 30S ribosomal protein 3, chloroplast precursor	*Ricinus communis*	19.8/4.41	20.4/7.79	116	11%	3	4.20	C	Chlo
2042	gi|13094963	Initiation factor eIF5-A	*Manihot esculenta*	18.5/5.09	17.8/5.60	73	10%	2	0.54	C	Nucl
1119	gi|402753	Translation elongation factor EF-G	*Glycine max*	37.1/5.12	77.9/5.04	69	4%	2	0.35	C	Chlo
2135	gi|218312	chloroplast elongation factor TuB (EF-TuB)	*Nicotiana sylvestris*	37.4/5.16	46.8/5.70	240	10%	3	0.05	C	Chlo
**Redox homeostasis**											
4015	gi|224091909	2-cys peroxiredoxin	*Populus trichocarpa*	32.5/5.35	28.9/6.84	191	19%	6	0.09	T	Chlo
1051	gi|220898265	Ascorbate peroxidase	*Ginkgo biloba*	29.1/4.77	27.7/5.81	118	11%	2	2.38	C	Chlo
0040	gi|294861514	Cytosolic ascorbate peroxidase 2	*Rubia cordifolia*	18.4/4.49	16.8/5.34	135	15%	1	5.36	C	Cyto
0041	gi|220898261	FeSOD	*Ginkgo biloba*	25.2/4.68	27.2/6.76	312	38%	5	1.94	C	Chlo
1028	gi|373842096	Peroxiredoxin	*Tamarix hispida*	19.8/4.74	17.6/6.08	83	10%	1	0.43	T	Cyto
5015	gi|45643751	Copper-zinc superoxide dismutase	*Citrullus lanatus*	20.2/5.75	15.1/5.05	120	17%	2	1.53	T	Cyto
**Unclassified**											
2128	gi|242079005	Hypothetical protein SORBIDRAFT_07g019320	*Sorghum bicolour*	35.5/5.09	46.7/4.83	195	8%	2	0.33	C	Chlo
4014	gi|297720697	Os01g0915900	*Oryza sativa* Japonica Group	26.6/5.74	28.3/8.75	57	8%	1	1.71	T	Nucl
5019	gi|224143607	Predicted protein	*Populus trichocarpa*	32.1/5.71	31.8/5.26	262	18%	4	1.53	T	Chlo
6117	gi|224080984	Predicted protein	*Populus trichocarpa*	34.9/5.62	42.5/5.69	113	15%	5	0.66	T	Chlo
1227	gi|116791600	Unknown	*Picea sitchensis*	40.9/5.05	21.0/8.48	143	7%	2	1.51	T	Cyto
1327	gi|116787373	Unknown	*Picea sitchensis*	44.9/4.86	65.8/5.69	159	7%	3	0.63	C	Chlo
0642	gi|148909901	Unknown	*Picea sitchensis*	57.6/4.59	63.4/5.12	118	5%	2	2.29	C	Chlo

^a^ Spot number corresponds to the differentially accumulated proteins indicated in Figure 2; ^b^ Accession number according to the National Center for Biotechnology Information (NCBI) database; ^c^ The names and species of the proteins obtained by MASCOT software; ^d^ The experimental mass (kDa) and pI of the identified proteins were calculated by PDQuest; ^e^ The theoretical mass (kDa) and pI values of the identified proteins were retrieved from the protein database; ^f^ MASCOT score after searching against the database; ^g^ The sequence coverage percentage of identified proteins; ^h^ Number of identified peptides (Peptide sequences were shown in Table S1); ^i^ The protein abundance ratio (YL/GL); ^j^ Type of extracts. T: total leaf proteins; C: chloroplast; ^k^ Sub-cellular localization. Chlo: Chloroplast, Cyto: Cytoplasmic, Nucl: Nuclear, Mito: Mitochondrial.

**Table 2:** Primers for qRT-PCR.

Gene	Primer Forward Sequence (5′–3′)	Primer Reverse Sequence (5′–3′)
*GAPDH*	GGTGCCAAAAAGGTGGTCAT	CAACAACGAACATGGGAGCAT
*SPDS*	ACATCTTCCACTTTGCTCTATTCCA	CGAGGGTCTTCATAGCCTACTGC
*HSP70*	ACTCAGAAGGGGCACGAACA	AAATCGCCTTCCTATCAACCG
*PSRP3*	TCATTGCCCACTTCATCCGC	CGCTCACTTCCTCTTCTGCTGC
*atpA*	TTATTGGGGACAGGCAGACCG	GGAGCGAGATATTGTAATGTAGCG
*GSA*	TGGCATCACTCCAGACCTTACA	GCAACCATCTCCATTATCTCCC
*CD4B*	AAGGCAGCCACAAATAGAACGG	CAAGACCCTCAGCAATAGCCG
*EF-Tu*	ATTTCCTGGAGACGATGTGCC	TCAGTCTGTCTCCGAGGAATGG

## Supplementary Materials

Supplementary materials can be found at www.mdpi.com/1422-0067/17/11/1794/s1.
