# Supplementary material for: Comparative Proteomic and Physiological Analysis Reveals the Variation Mechanisms of Leaf Coloration and Carbon Fixation in a Xantha Mutant of Ginkgo biloba L
Source: Int J Mol Sci. 2016 Oct 27;17(11):1794. doi: 10.3390/ijms17111794 (PMC5133795; doi:10.3390/ijms17111794)
Supplement: Supplementary file 1 [file ijms-17-01794-s001.pdf]

Supplementary Materials: Comparative Proteomic and Physiological Analysis Reveals the Variation Mechanisms of Leaf Coloration and Carbon Fixation in a Xantha Mutant of *Ginkgo biloba* L.

Xinliang Liu, Wanwen Yu, Guibin Wang, Fuliang Cao, Jinfeng Cai and Huanli Wang

Table S1. Peptide sequences of the proteins identified by MASCOT. Matched peptides are shown in red.

**Spot 0561 (A4-1.mht)**				
MATAVSTVGA	ATRAPLNLNG	SSAGASVPTS	GFLGSSLKKH	TNVRFPSSSR
TTSMTVKAAE	NEEKNTDKWA	HLAKDFSDDQ	LDIRRGKGMV	DSLFQAPADA
GTHVPIQSSF	EYESQGLRKY	DIDNMLGDLY	IAPAFMDKLV	VHITKNFLNL
PNIKIPLILG	VWGGKGQGKS	FQCELVFAKL	GINPIMMSAG	ELESGNAGEP
AKLIRQRYRE	AADLIAKGKM	CALFINDLEP	GAGRMGGTTQ	YTVNNQMVNA
TLLNIADNPT	NVQLPGMYNK	QDNARVPIIV	TGNDFSTLYA	PLIRDGRMEK
FYWAPTREDR	IGVCTGIFKT	DKVPAEHVVK	LVDAFPGQSI	DFFGALRARV
YDDEVRKWVN	SVGVDNVGKK	LVNSKDGPPV	FEQPEMTLQK	LMEYGNMLVQ
EQENVKRVQL	ADQYMSSAAL	GDANKDAIDR	GTFFGKAAQQ	VSLPVAQGCT
DPEAKNYDPT	ARSDDGSCTY	NL		
**Spot 2132 (A9-1.mht)**				
MAPQAYPTAG	QTTTVCVTGA	AGFMASWLVK	RLLEKGYIVH	ATVRDPENKA
KVSHLLNLPG	ATDRLKLFRA	ELCEDGSFDA	AVAGCNGVFH	VATPTEFMPK
DPENDLIKPA	IEGTLNVLKS	CTKVDSIKRV	VVTSSAATVS	INNSSEQNQY
IDESCWTDVN	FLTSQKPPGW	AYPVSKTLAE	QAALKYAEEH	SLDVVTVIPV
LVVGPAVTPT	VPSSVELALS	LITGDEFKMG	ALKGMQFVSG	SISLVHIDDV
CSAQIFLMEK	PSAQGRYICF	PVNTGIPQLA	EFLSKRYPQY	KVPTKFDDVP
ATPKLTISSQ	KLLDCGFSFK	YGIEDIYDQA	IEYMKTKGLL	TC
**Spot 7023 (A10-1.mht)**				
YKVTKGMAEK	YGDLRVLDTP	IAENSFTGMG	VGAAMTGLRP	VIEGMNMGFL
LLAFNQISNN	CGMLHYTSGG	QFTIPVVIRG	PGGVGRQLGA	EHSQRLESYF
QSVPGLQMVA	CSTPYNAKGL	MKAAIRSENP	VILFEHVLLY	NLKEKIPDEE
YVCCLEEAEM	VRPGADVTIL	TYSRMRYHVM	QAAKTLVNKG	YDPEIIDIRS
LKPFDLHTIG	NSIKKTHRVL	IVEECMRTGG	IGASLRAAII	ENFWDYLDAP
IMCLSSQDVP	TPYAGTLEDW	TVVQPPQIVS	AVEQICQ	
**Spot 0218 (A16-1.mht)**				
METSITCYTR	GAFLPNISSS	HSMSFVSPSS	ISPSFNSKIL	KSSSLFGESL
RTVPKSSLKV	SKSKNALPVT	KCEIGESLEE	FLIKATPDKG	LRALLISMGE
ALRTIAFKVR	TASCGGTACV	NSFGDEQLAV	DMLANNLLFE	ALRYSHFCKY
ACSEEVPELQ	DMGGPVEGGF	SVAFDPLDGS	SIVDTNFTVG	TIFGVWPGDK
LTGVTGRDQV	AAAMGIYGPR	TTYVLALKDV	PGTHEFLLLD	EGKWQHVKET
TEIGEGKLFS	PGNLRATFDN	PEYNKLIDYY	VKEKYTLRYT	GGRVPDVNQI
IVKEKGIFTN	VLSPSAKAKL	RLLFEVAPLG	FLIEKAGGFS	SDGHQSVLEK
VIENLDDRTQ	VAYGSKNEII	RFEETLYGSS	RLKGAVPVGA	AA
**Spot 0846 (A20-1.mht)**				
MAGKGDGPAI	GIDLGTTYSC	VGVWQHDRVE	IIANDQGNRT	TPSYVAFTDT
ERLIGDAAKN	QVAMNPINTV	FDAKRLIGRR	FSDASVQSDA	KLWPFKVIPG
PGDKPMIGVQ	YRGEDKQFSA	EEISSMVLNK	MKETAEAYLG	TTIKNAVVTV
PAYFNDSQRQ	ATKDAGVISG	LNVMRIINEP	TAAAIAYGLD	KKSTSVGEKN
VLIFDLGGGT	FDVSLLTIEE	GIFEVKATAG	DTHLGGEDFD	NRMVNHFVQE
FKRKNKKDIS	GNPRALRRLR	TACERAKRTL	SSTAQTTIEI	DSLFEGIDFY
TTITRARFEE	LNMDLFRKCM	EPVEKCLRDA	KMDKSTVHDV	VLVGGSTRIP

RVQQLLQDFF	NGKELCKSIN	PDEAVAYGAA	VQAAILTGEG	NEKVQDLLLL
DVTPLSQGLE	TAGGVMTVLI	PRNTTIPTKK	EQVFSTYSDN	QPGVLIQVYE
GERARTKDNN	LLGKFELSGI	PPAPRGVPQI	TVCFDIDANG	ILNVSAEDKT
TGQKNKITIT	NDKGRLSKED	IEKMVQEAEK	YKAEDEEHKK	KVDAKNSLEN
YAYNMRNTIK	DDKIASKLPE	ADKKKIEDAI	DGAITWLDNN	QLAEADEFDD
KMKELEGICN	PIIAKMYQGA	GAEMPGGMDE	DTPASTAGGS	SGPGPKIEEV
D				
**Spot 0322 (A23-1.mht)**				
MASASVVKCM	SAPQYGIQRT	SMAGFSASSV	HGVKLGSMHA	ESARSHRVLM
TAMLPPKAKA	VEKVKTQFDP	RETHVQVTHS	MPPQKMEIFK	SLEEWAENNI
LVHLKPVERS	WQPQDFLPDP	ASEGFMDEVK	ELRNRAQEIP	DDYFVCLVGD
MITEEALPTY	QTMLNTLDGV	RDDTGASPTS	WAVWTRAWTA	EENRHGDLLN
KYLYLSGRVD	MRQIEKTIQY	LIGSGMDPRT	ENSPYLGFVY	TSFQERATFI
SHGNTARHAK	DYGDLKLAQI	CGTIASDEKR	HETAYTKIVE	KLFEVDPDST
VLAFEDMMRK	KISMPAHLMY	DGQDDNLFHH	FSAVAQRLGV	YTAKDYADIL
EFLVKRWNVE	KIEGLSSEGR	KAQEFVCKLA	PRIRRLEERA	QQRAKQGEII
PFSWIFNREV	SM			
**Spot 2646 (C2-1.mht)**				
MATAVSTVGA	ATRAPLNLNG	SSAGASVPTS	GFLGSSLKKH	TNVRFPSSSR
TTSMTVKAAE	NEEKNTDKWA	HLAKDFSDDQ	LDIRRGKGMV	DSLFQAPADA
GTHVPIQSSF	EYESQGLRKY	DIDNMLGDLY	IAPAFMDKLV	VHITKNFLNL
PNIKIPLILG	VWGGKGQGKS	FQCELVFAKL	GINPIMMSAG	ELESGNAGEP
AKLIRQRYRE	AADLIAKGKM	CALFINDLEP	GAGRMGGTTQ	YTVNNQMVNA
TLLNIADNPT	NVQLPGMYNK	QDNARVPIIV	TGNDFSTLYA	PLIRDGRMEK
FYWAPTREDR	IGVCTGIFKT	DKVPAEHVVK	LVDAFPGQSI	DFFGALRARV
YDDEVRKWVN	SVGVDNVGKK	LVNSKDGPPV	FEQPEMTLQK	LMEYGNMLVQ
EQENVKRVQL	ADQYMSSAAL	GDANKDAIDR	GTFFGKAAQQ	VSLPVAQGCT
DPEAKNYDPT	ARSDDGSCTY	NL		
**Spot 3841 (C5-1.mht)**				
MASITASHHF	VSRSQTSLDT	KSTLSQIGLR	NHTLTHNGLR	AVNKLDGLQS
RTNTKVTPKM	ASRTETKRPG	CSATIVCGKG	MNLIFVGTEV	GPWSKTGGLG
DVLGGLPPAL	AARGHRVMTI	SPRYDQYKDA	WDTSVAVEVK	VGDSIEIVRF
FHCYKRGVDR	VFVDHPMFLE	KVWGKTGSKI	YGPKAGLDYL	DNELRFSLLC
QAALEAPKVL	NLNSSNYFSG	PYGEDVLFIA	NDWHTALIPC	YLKSMYQSRG
IYLNAKVAFC	IHNIAYQGRF	SFSDFPLLNL	PDEFRGSFDF	IDGYEKPVKG
RKINWMKAGI	LESHRVVTVS	PYYAQELVSA	VDKGVELDSV	LRKTCITGIV
NGMDTQEWNP	ATDKYTDVKY	DITTVMDAKP	LLKEALQAAV	GLPVDKKIPL
IGFIGRLEEQ	KGSDILVAAI	HKFIGLDVQI	VVLGTGKKEF	EQIEQLEVL
YPGKVKGVAK	FNVPLAHMIT	AGADFMLVPS	RFEPCGLIQL	HAMRYGTVPI
CASTGGLVDT	VKEGYTGFHM	GAFNVECDVV	DPADVLKIVT	TVARALAVYG
TLAFAEMIKN	CMSEELSWKE	PAKKWETLLL	GLGASGSEPG	VEGEEIAPLA
KENVATP				
**Spot 2651 (C7-1.mht)**				
MATAVSTVGA	ATRAPLNLNG	SSAGASVPTS	GFLGSSLKKH	TNVRFPSSSR
TTSMTVKAAE	NEEKNTDKWA	HLAKDFSDDQ	LDIRRGKGMV	DSLFQAPADA
GTHVPIQSSF	EYESQGLRKY	DIDNMLGDLY	IAPAFMDKLV	VHITKNFLNL
PNIKIPLILG	VWGGKGQGKS	FQCELVFAKL	GINPIMMSAG	ELESGNAGEP

AKLIRQRYRE	AADLIAKGKM	CALFINDLEP	GAGRMGGTTQ	YTVNNQMVNA
TLLNIADNPT	NVQLPGMYNK	QDNARVPIIV	TGNDFSTLYA	PLIRDGRMEK
FYWAPTREDR	IGVCTGIFKT	DKVPAEHVVK	LVDAFPGQSI	DFFGALRARV
YDDEVRKWVN	SVGVDNVGKK	LVNSKDGPPV	FEQPEMTLQK	LMEYGNMLVQ
EQENVKRVQL	ADQYMSSAAL	GDANKDAIDR	GTFFGKAAQQ	VSLPVAQGCT
DPEAKNYDPT	ARSDDGSCTY	NL		
**Spot 2130 (C15-1.mht)**				
MAKEGTKVPR	IKLGSQGLEV	SAQGLGCMGM	SAFYGPPKPE	PDMIQLIHHA
INSGITLLDT	SDVYGPHTNE	ILLGKALKGG	TRERVVLATK	FGIVLGDEKK
AEGKRAVHGD	PAYVRAACEA	SLKRLDIDCI	DLYYQHRVDT	RVPIEITVGE
LKKLVEEGKL	KYIGLSEASA	STIRRAHAVH	PITAVQLEWS	LWSRDVEEEI
IPTCRELGIG	IVAYSPLGRG	FLSSGPKLLE	DMSNEDYRKY	LPRFQAENLE
NNKNLYERIC	EMAVRKGCTP	SQLALAWVHH	QGNDVCPIPG	TTKIENLNQN
MKPCPSS				
**Spot 1327 (C17-1.mht)**				
MLRLQRILRQ	AKPASRVLAL	NLSRQYAKDV	KFGADARALM	LQGVDLLADA
VAVTMGPKGR	TVIIEQSWGS	PKVTKDGVTV	AKAIELKDKY	KNIGAKLVQD
VANNTNEEAG	DGTTTATVLA	RSIAKEGFEK	ISKGANPIEI	RRGVMLAVDA
VIAELKKQSK	PVTTPEEIAQ	VATISANGDQ	EIGKIISDAM	KKVGRKGVIT
VKDGKTLHDE	LEIIEGMKFD	RGYISPYFIN	TAKGQKCEFQ	DAYLLLSEKK
ISNVQPIVPA	LEIANAHRKP	LVIIAEDVDG	EALSTLVLNR	LKVGLQVVAV
KAPGFGDNRK	NQLKDMAVSS	GGVVFGEEGL	TLNLEDIQPH	DFGKVGEVIV
TKDDTMLLKG	KGDKALIEKR	IQEIHDQLET	TNSEYEKEKL	NERLAKLSDG
VAVLKVGGTS	DVEVNEKKDR	VTDALNATRA	AVEEGIVLGG	GCALLRCIPA
LDSINPVNED	QKIGKLLC			
**Spot 0740 (C18-1.mht)**				
MTMITPSSNT	THYRESWYAC	RYRSGIPGST	HASVASRRVL	SSLLRSSSGR
SAAKLGNRNP	RLPSPSPARH	AAPCSYLLGR	VAEYATSSPA	SSAAPSSAPA
KDEGKKTYDY	GGKGAIGRVC	QVIGAIVDVR	FEDQEGLPPI	MTSLEVQDHP
TRLVLEVSHH	LGQNVVRTIA	MDGTEGLVRG	RKVLNTGAPI	TVPVGRATLG
RIMNVLGEPI	DERGEIKTEH	YLPIHRDAPA	LVDLATGQEI	LATGIKVVDL
LAPYQRGGKI	GLFGGAGVGK	TVLIMELINN	VAKAHGGFSV	FAGVGERTRE
GNDLYREMIE	SGVIKLGEKQ	SESKCALVYG	QMNEPPGARA	RVGLTGLTVA
EYFRDAEGQD	VLLFIDNIFR	FTQANSEVSA	LLGRIPSAVG	YQPTLASDLG
ALQERITTTK	KGSITSVQAI	YVPADDLTDP	APATTFAHLD	ATTVLSRQIS
ELGIYPAVDP	LDSTSRMLSP	HILGEEHYNT	ARGVQKVLQN	YKNLQDIIAI
LGMDELSEDD	KLTVARARKI	QRFLSQPFHV	AEIFTGAPGK	YVDLKENINS
FQGLLDGKYD	DLSEQSFYMV	GGIDEVVAKA	EKIAKESAA	
**Spot 2649 (C20-1.mht)**				
MRTNPLVLGV	SALVEKNVGR	IAQIIGPVLD	VSFPPGNMPN	IYNSLIVKGQ
GTAGQEIQVT	CEVQQLLGNH	KVRAVAMSAT	DGLTRGMRVI	DTGAPLSVPV
GGATLGRIFN	VLGEPVDNLG	PVDARITSPI	HRSAPAFTEL	DTKLSIFETG
IKVVDLLAPY	RRGGKIGLFG	GAGVGKTVLI	MELINNIAKA	HGGVSVFGGV
GERTREGNDL	YMEMKESGVI	DEQNISESKV	ALVYGQMNEP	PGARMRVGLT
ALTMAEYFRD	VNEQDVLSFI	DNIFRFVQAG	SEVSALLGRM	PSAVGYQPTL
ATEMGSLQER	ITSTKRGSIT	SIQAVYVPAD	DLTDPAPATT	FAHLDATTVP
SRGLAAKGIY	PAVDPLDSTS	TMLQPWIVGE	EHYETAQGVK	QTLQRYKELQ

DIIAIPGLDE	LSEEDRLIVA	RARKIERFLS	QPFFVAEVFT	GFPGKYVGLM
ETIRGFQMIL	SGELEQSFYL	VGNIDEATAK	AMNSKTES	
**Spot 1051 (C22-1.mht)**				
MGKSYPTVSE	EYKAAIEKCR	RKLRALIADK	NCAPIMVRLA	WHGAGTYDVK
TNTGGPFGTI	RYSAELAHGA	NNGLIIAVRL	LEPIKAQFPI	ISYADLYQLA
GVVAVEITGG	PDISFHPGRK	DKLEHEAPEE	GRLPDATKGS	DHLRDVFGHM
GLSDKDIVAL	SGAHTLGRCH	KERSGFEGPW	TANPLIFDNS	YFTELVTGEK
EGLLQLPSDK	ALLIDPKFAV	YVHKYAQDED	AFFADYAESH	QKLSELGFAE
A				
**Spot 3514 (D3-1.mht)**				
MRTNPLVLGV	SALVEKNVGR	IAQIIGPVLD	VSFPPGNMPN	IYNSLIVKGQ
GTAGQEIQVT	CEVQQLLGNH	KVRAVAMSAT	DGLTRGMRVI	DTGAPLSVPV
GGATLGRIFN	VLGEPVDNLG	PVDARITSPI	HRSAPAFTEL	DTKLSIFETG
IKVVDLLAPY	RRGGKIGLFG	GAGVGKTVLI	MELINNIAKA	HGGVSVFGGV
GERTREGNDL	YMEMKESGVI	DEQNISESKV	ALVYGQMNEP	PGARMRVGLT
ALTMAEYFRD	VNEQDVLSFI	DNIFRFVQAG	SEVSALLGRM	PSAVGYQPTL
ATEMGSLQER	ITSTKRGSIT	SIQAVYVPAD	DLTDPAPATT	FAHLDATTVP
SRGLAAKGIY	PAVDPLDSTS	TMLQPWIVGE	EHYETAQGVK	QTLQRYKELQ
DIIAIPGLDE	LSEEDRLIVA	RARKIERFLS	QPFFVAEVFT	GFPGKYVGLM
ETIRGFQMIL	SGELEQSFYL	VGNIDEATAK	AMNSKTES	
**Spot 2339 (D4-1.mht)**				
MADAEDIQPL	VCDNGTGMVK	AGLAGDDAPR	AVFPSIVGRP	RHTGVMVGMG
QKDAYVGDEA	QSKRGILTLK	YPIEHGIVSN	WDDMEKIWHH	TFYNELRVAP
EEHPILLTEA	PLNPKANREK	MTQIMFETFT	LHVPAMYVAI	QAVLSLYASG
RTTGIVLDSG	DGVSHTVPIY	EGYALPHAIL	RLDRAGRDLT	DSLMKILTER
GYSVTTPAER	EIAADIKEKL	AYVRLDYEQE	LETAKSSSSV	EKSYELPDGQ
VITIGAERFR	CPEVMFQPSL	IGMEAPGIHE	TTYNSIMKCD	VDIRKDLYGN
IVLSGGSTMF	PGIRRPMSKE	DHRAAPSSMK	IKVVAPPERK	YSVWIGGSIL
ASLSTFQQVW	ISRAEYEESG	PAIVHRKCF		
**Spot 5144 (D9-1.mht)**				
MATAVSTVGA	ATRAPLNLNG	SSAGASVPTS	GFLGSSLKKH	TNVRFPSSSR
TTSMTVKAAE	NEEKNTDKWA	HLAKDFSDDQ	LDIRRGKGMV	DSLFQAPADA
GTHVPIQSSF	EYESQGLRKY	DIDNMLGDFY	IAPAFMDKLV	VHITKNFLNL
PNIKIPLILG	VWGGKGQGKS	FQCELVFAKL	GINPIMMSAG	ELESGNAGEP
AKLIRQRYRE	AADLIAKGKM	CALFINDLEP	GAGRMGGTTQ	YTVNNQMVNA
TLMNIADNPT	NVQLPGMYNK	QDNARVPIIV	TGNDFSTLYA	PLIRDGRMEK
FYWAPTREDR	IGVCTGIFKT	DKVPAEHVVK	LVDAFPGQSI	DFFGALRARV
YHDEVRKWVN	SVGVDNVGKK	LVNSKDGPPV	FEQPEMTLQK	LMEYGNMLVQ
EQENVKRVQL	ADQYMSSAAL	GDANKDAIDR	GTFFG	
**Spot 0641 (D10-1.mht)**				
MASTFTATSS	IGSMVAPNGH	KSDKKLISKL	SSSSFGRRQS	VCPRPRRSSS
AIVCAAKELH	FNKDGTTIRR	LQAGVNKLAD	LVGVTLGPKG	RNVVLESKYG
SPRIVNDGVT	VAREVELEDP	VENIGAKLVR	QAAAKTNDLA	GDGTTTSVVL
AQGFIAEGVK	VVAAGANPVL	ITRGIEKTAK	ALVTELKKMS	KEVEDSELAD
VAAVSAGNND	EIGNMIAEAM	SKVGRKGVVT	LEEGKSAENN	LYVVEGMQFD
RGYISPYFVT	DSEKMSVEFD	NCKLLLVDKK	ITNARDLVGV	LEDAIRGGYP
ILIIAEDIEQ	EALATLVVNK	LRGTLKIAAL	RAPGFGERKS	QYLDDIAILT

GATVIREEVG	LSLDKAGKEV	LGNASKVVLT	KETSTIVGDG	STQDAVKKRV
TQIKNLIEQA	EQDYEKEKLN	ERIAKLSGGV	AVIQVGAQTE	TELKEKKLRV
EDALNATKAA	VEEGIVVGGG	CTLLRLASKV	DAIKATLDND	EEKVGADIVK
RALSYPLKLI	AKNAGVNGSV	VSEKVLSNDN	VKFGYNAATG	KYEDLMAAGI
IDPTKVVRCC	LEHAASVAKT	FLMSDCVVVE	IKEPEPVPVG	NPMDNSGYGY
**Spot 8221 (D13-1.mht)**				
MGDAEEIQPL	VCDNGSGMVK	AGFAGDDAPR	AVFPSIVGRP	RHTGVMVGMG
QKDAYVGDEA	QSKRGILTLK	YPIDHGIVTN	WDDMEKIWHH	TFYNELRVAP
EEHPVLLTEA	PLNPKANREK	MTQIMFETFN	VPAMYVAIQA	VLSLYASGRT
TGIVLDSGDG	VTHTVPIYEG	YALPHAILRL	DLAGRDLTDA	LMKILTERGY
TFTTTAEREI	VRDMKEKLAY	VSLDFEQEME	TAKTSSSLEK	SYELPDGQVI
TIGAERFRCA	EVLFQPSLIG	MEAAGIHETT	YNSIMKCDVD	IRKDLYGNIV
LSGGSTMFPG	IADRMSKEIT	SLAPSSMKIK	VVAPPERKYS	VWIGGSILAS
LSTFQQMWIS	KSEYEESGPS	IVHRKCF		
**Spot 0223 (D18-1.mht)**				
MATAVSTVGA	VNSVPLSLHG	SSSGAPAPTS	AFFGSNLKKV	NPSLTHGRVQ
TGSFKVMAVD	LDETKQTKTD	RWQLHKDTSD	DQQDIVRGKG	LVDPLFQAPM
GDGTHEAVLN	SYEYISQGLR	DYGMENKMDG	FYIAPEFMDK	LVVHITKNYM
DLPNIKVPLI	LGIWGGKGQG	KTFQCELVFR	KMGINPIMMS	AGELESGNAG
EPAKLIRQRY	REAADIIAKG	KMCCLFINDL	DAGAGRMGGT	TQYTVNNQMV
NATLMNIADN	PTNVQLPGMY	NKQENARVPI	IVTGNDFSTL	YAPLIRDGRM
EKFYWAPTRS	DRIGVCTGIF	MTDNVAVQDI	VKLVDAFPGQ	SIDFFGALRA
RVYDDEVRKW	ISDIGVDKVG	KRLVNSAEGA	PTFEQPKMTL	EKLMDYGNML
VQEQENVKRV	QLADKYLSEA	ALGDANVDAI	KTGKF	
**Spot 3940 (D20-1.mht)**				
MARALVQSTS	IPSSVAGERT	TKFNGSGKTK	RAVTMLCNAQ	SSSLTLRDFT
GLRGCNAIDT	LVRSGETLQS	KVAAATYVRR	PRGCRFVPKA	MFERFTEKAI
KVIMLAQEEA	RRLGHNFVGT	EQILLGLIGE	GTGIAAKVLK	SMGINLKDAR
VEVEKIIGRG	SGFVAVEIPF	TPRAKRVLEL	SLEEARQLGH	NYIGSEHLLL
GLLREGEGVA	ARVLENLGAD	PSNIRTQVIR	MVGESNEAVG	ASVGGGTSGQ
KMPTLEEYGT	NLTKLAEEGK	LDPVVGRQPQ	IERVTQILGR	RTKNNPCLIG
EPGVGKTAIA	EGLAQRIANG	DVPETIEGKK	VITLDMGLLV	AGTKYRGEFE
ERLKKLMEEI	KQSDEIILFI	DEVHTLIGAG	AAEGAIDAAN	ILKPALARGE
LQCIGATTLD	EYRKHIEKDP	ALERRFQPVK	VPEPTVDETI	QILKGLRERY
EIHHKLRYTD	EDLVAAAQLS	YQYISDRFLP	DKAIDLIDEA	GSRVRLRHAQ
LPEEAKELEK	ELRQITKEKN	EAVRGQDFEK	AGELRDREMD	LKAQITALID
KNKEVSKAES	EAADTGPLVT	EADIQHIVSS	WTGIPVEKVS	TDESDRLLKM
EETLHTRIIG	QDEAVKAISR	AIRRARVGLK	NPNRPIASFI	FSGPTGVGKS
ELAKALAAYY	FGSEEAMIRL	DMSEFMERHT	VSKLIGSPPG	YVGYTEGGQL
TEAVRRRPYT	VVLFDEIEKA	HPDVFNMMLQ	ILEDGRLTDS	KGRTVDFKNT
LLIMTSNVGS	SVIEKGGRRI	GFDLDLDEKD	SSYNRIKSLV	TEELKQYFRP
EFLNRLDEMI	VFRQLTKLEV	KEIADIMLKE	VFERLKVKEI	ELQVTERFRD
RVVDEGYNPS	YGARPLRRAI	MRLLEDSMAE	KMLANEIKEG	DSVIVDVDSD
GNVTVLNGSS	GTPSDPAPEP	IPV		
**Spot 2627 (E3-1.mht)**				
MATAVSTVGA	ATRAPLNLNG	SSAGASVPTS	GFLGSSLKKH	TNVRFPSSSR
TTSMTVKAAE	NEEKNTDKWA	HLAKDFSDDQ	LDIRRGKGMV	DSLFQAPADA

GTHVPIQSSF	EYESQGLRKY	DIDNMLGDLY	IAPAFMDKLV	VHITKNFLNL
PNIKIPLILG	VWGGKGQGKS	FQCELVFAKL	GINPIMMSAG	ELESGNAGEP
AKLIRQRYRE	AADLIAKGKM	CALFINDLEP	GAGRMGGTTQ	YTVNNQMVNA
TLLNIADNPT	NVQLPGMYNK	QDNARVPIIV	TGNDFSTLYA	PLIRDGRMEK
FYWAPTREDR	IGVCTGIFKT	DKVPAEHVVK	LVDAFPGQSI	DFFGALRARV
YDDEVRKWVN	SVGVDNVGKK	LVNSKDGPPV	FEQPEMTLQK	LMEYGNMLVQ
EQENVKRVQL	ADQYMSSAAL	GDANKDAIDR	GTFFGKAAQQ	VSLPVAQGCT
DPEAKNYDPT	ARSDDGSCTY	NL		

Spot 2848 (E7-1.mht)

MASSTAQIHA	LGATQFATGR	SRTVFFGQRV	SCRTVPFGLK	LKKSRGNAGG
TRLRIVAEKV	VGIDLGTTNS	AVAVMEGGKP	TIVTNAEGQR	TTPSVVAYTK
NGDRLVGQIA	KRQAVVNPEN	TFFSVKRFIG	RKMSEVDEES	KQVSYNVIRD
ENGNVKLDCP	AIGKQFAAEE	ISAQVLRKLV	DDASKFLNDN	VSKAVVTVPA
YFNDSQRTAT	KDAGRIAGLE	VLRIINEPTA	ASLAYGFERK	NNETILVFDL
GGGTFDVSVL	EVGDGVFEVL	STSGDTHLGG	DDFDKRVVDW	LASNFKKDEG
IDLLKDKQAL	QRLTETAEKA	KMELSSLTQT	NISLPFITAT	ADGPKHIETT
LTRAKFEELC	SDLLDRLKTP	VQNSLRDAKL	SFSDIDEVIL	VGGSTRIPAV
QEVVKKLTGK	DPNVSVNPDE	VVALGAAVQA	GVLAGDVSDI	VLLDVTPLSL
GLETLGGVMT	KIIPRNTTLP	TSKSEVFSTA	ADGQTSVEIN	VLQGEREFVR
DCKSLGSFRL	DGIPPAPRGV	PQIEVKFDID	ANGILSVAAV	DKGTGKKQDI
TITGASTLPN	DEVDRMVKEA	ERFAQEDKEK	RDAIDTKNQA	YSVVYQTEKQ
LKDLGDKIPG	SVKEKVEAKL	GELKDAISGG	STQAMKDAMA	ALNQEVMQIG
QSLYNQPGAA	PGAGPAPGGS	AEPSESSSSS	GKGPDGDVID	ADFTDSN

Spot 0642 (E11-1.mht)

MAKIVAFDEE	SRRAIERGVN	ALADAVRVTL	GPRGRNVLIE	KKFGVPDIVS
DGITVAKAIE	LGDPLENTGA	RLIQEVAAKT	NDVAGDGTTT	AAVLAQAMIQ
EGLKNVAAGA	NPVALRRGID	KTVQYLVKEI	ESLAKPVEGS	AIEQVATVSA
GNDKEVGEMI	ALAMDKVTKD	GVITVEESKS	LTTELEVVEG	MQIDRGYISP
YFITDSERMT	VELDNARVLI	TDKKISAIQD	IVSVLEKVAR	SGQPLLIIAE
DIDGEALATL	VVNKARGVLN	VAAIKAPGFG	ERRKAMLQDI	AILTGGQLIS
EEIGLNLETA	TVEMLGTATK	VTINKDSTTI	VSGSGHQGEI	QQRVEQLRKQ
LAETDSEYDQ	EKLQERIAKL	AGGVAVIKVG	AATETELKSR	KLRIEDALNA
TKAAVDEGIV	PGGGTTLIHL	VKKVEQLAAT	FSIEEEKLGA	KIVARALEAP
LRQIANNSGV	EGSVIVEQVR	NSDSNIGYNA	LTGNFEDLIV	AGILDPAKVV
RSSLQNAGSI	AGMVITTEVL	VVEKPEPKPA	MPDMDGMGGM	GGMGGMGGMG
GMGGMGMM				

Spot 2133 (E19-1.mht)

MFIESFKVES	PNVRYGAGEI	ESEYRYDTTE	LVHESHDGAS	KWVVRPKSVN
YHFKTNTTVP	KLGVMLVGWG	GNNGSTLMAG	VIANREGISW	ATKDKVQQAN
YFGSLTQAST	IRVGSYNGEE	IYAPFKSLLP	MVNPDDLVFG	GWDISSMNLA
DAMTRTKVLD	IDLQKQLRPY	MESIVPLPGI	YDPDFIAANQ	GSRANNVIKG
TKKEQMEQII	KDIREFKEKN	KVDKVVVLWT	ANTERYSNVS	VGLNDTMENL
LASVDKNEAE	ISPSTLYAIA	CVMEGVPFIN	GSPQNTFVPG	LIDLAIKNNC
LIGGDDFKSG	QTKMKSVLVD	FLVGAGIKPT	SIVSYNHLGN	NDGMNLSAPQ
TFRSKEISKS	NVVDDMVSSS	AILYKPGEHP	DRVVVIKYVP	YVGDSKRAMD
EYTSEIFMGG	KSTIVLHNTC	EDSLLAAPII	LDLVLLAELS	TRIQLKAEGE
DKFHSFHPVA	TILSYLTKAP	LVPPGTPVVN	ALAKQRAMLE	NIMRACVGLA

PENNMILEYK				
		**Spot 2134 (E22-1.mht)**		
MPMVTIRPDE	ISSIIRKQIE	QYNQEVEVAN	IGTVLRVGDG	IARIHGLDEV
MAGELVEFVD	GTVGIAPNLE	SNDVGVVLMG	DGLMIQEGSS	VRATGKIAQI
PVSDAYLGRV	VNALAQPIDG	RGQISASESR	LIESSAPGII	LRRSVYEPLQ
TGLIAIDSMI	PIGRGQRELI	IGDRQTGKTA	VATDTIINQK	GQDVICVYVA
IGQKASSVAQ	VVNTSQERGA	MEYTIVVAET	ADSPATLQYL	APYTGAALAE
YFMYQEQHTP	IIYDDPSKQA	RAYRQMSLLL	RRPPGREAYP	GDVFYLHSRL
LERAAKLSSQ	LGEGSMTALP	IVETQAGDVS	AYIPTNVISI	TDGQIFLSAD
LFNAGIQPAI	DVGISVSRVG	SAAQIKAMKQ	VAGKLKLELA	QFAELEAFAQ
FASDLDKATQ	NQLARGQRLR	ELLKQSQSAP	LTVEEQIATI	YTGANGYLDI
SEIVQVRKFL	VQLREYLITN	KPQFGEIIRS	TRTFTEQAEA	LLKEAIKEHI
EIFLTSRTEE	NI			
		**Spot 3719 (E23-1.mht)**		
MAGKGDGPAI	GIDLGTTYSC	VGVWQHDRVE	IIANDQGNRT	TPSYVAFTDT
ERLIGDAAKN	QVAMNPINTV	FDAKRLIGRR	FSDASVQSDA	KLWPFKVIPG
PGDKPMIGVQ	YRGEDKQFSA	EEISSMVLNK	MKETAEAYLG	TTIKNAVVTV
PAYFNDSQRQ	ATKDAGVISG	LNVMRIINEP	TAAAIAYGLD	KKSTSVGEKN
VLIFDLGGGT	FDVSLLTIEE	GIFEVKATAG	DTHLGGEDFD	NRMVNHFVQE
FKRKNKKDIS	GNPRALRRLR	TACERAKRTL	SSTAQTTIEI	DSLFEGIDFY
TTITRARFEE	LNMDLFRKCM	EPVEKCLRDA	KMDKSTVHDV	VLVGGSTRIP
RVQQLLQDFF	NGKELCKSIN	PDEAVAYGAA	VQAAILTGEG	NEKVQDLLLL
DVTPLSQGLE	TAGGVMTVLI	PRNTTIPTKK	EQVFSTYSDN	QPGVLIQVYE
GERARTKDNN	LLGKFELSGI	PPAPRGVPQI	TVCFDIDANG	ILNVSAEDKT
TGQKNKITIT	NDKGRLSKED	IEKMVQEAEK	YKAEDEEHKK	KVDAKNSLEN
YAYNMRNTIK	DDKIASKLPE	ADKKKIEDAI	DGAITWLDNN	QLAEADEFDD
KMKELEGICN	PIIAKMYQGA	GAEMPGGMDE	DTPASTAGGS	SGPGPKIEEV
D				
		**Spot 1119 (E24-1.mht)**		
MSADGDAKRS	VPLKDYRNIG	IMAHIDAGKT	TTTERILYYT	GRNYKIGEVH
EGTATMDWME	QEQERGITIT	SAATTTFWNK	HRINIIDTPG	HVDFTLEVER
ALRVLDGAIC	LFDSVAGVEP	QSETVWRQAD	KYGVPRICFV	NKMDRLGANF
YRTRDMIVTN	LGAKPLVIQL	PIGSEDNFKG	VIDLVRNKAI	VWSGEELGAK
FDIVDIPEDL	QEQAQDYRAQ	MIENIVEFDD	QAMENYLEGI	EPDEETIKKL
IRKGTISASF	VPVMCGSAFK	NKGVQPLLDA	VVDYLPSPLD	LPAMKGSDPE
NPEATIERLA	SDDEPFAGLA	FKIMSDPFVG	SLTFVRVYAG	KLGAGSYVLN
ANKGKKERIG	RLLEMHANSR	DDVKVALAGD	IIALAGLKDT	ITGETLCDPD
NPIVLERMDF	PDPVIKVAIE	PKTKADVDKM	ATGLIKLAQE	DPSFHFSRDE
EINQTVIEGM	GELHLEIIVD	RLKREFKVEA	NVGAPQVNYR	ESISKISEVK
YVHKKQSGGQ	GQFADITVRF	EPMDPGSGYE	FKSEIKGGAV	PREYIPGVMK
GLEECMSNGV	LAGFPVVDVR	AVLTDGSYHD	VDSSVLAFQL	AARGAFREGI
RKAGPRMLEP	IMKVEVVTPE	EHLGDVIGDL	NSRRGQINSF	GDKPGGLKVV
DSLVPLAEMF	QYVSTLRGMT	KGRASYTMQL	AMFDVVPQHI	QNQLATKEQE
VAA				
		**Spot 2522 (F1-1.mht)**		
MSPQTETKAS	VGFKAGVKEY	KLTYYTPEYQ	TKDTDILAAF	RVTPQPGVPP
EEAGAAVAAE	SSTGTWTTVW	TDGLTSLDRY	KGRCYRIERV	VGEKDQYIAY

VAYPLDLFEE	GSVTNMFTSI	VGNVFGFKAL	RALRLEDLRI	PPAYVKTFQG
PPHGIQVERD	KLNKYGRPLL	GCTIKPKLGL	SAKNYGRAVY	ECLRGGLDFT
KDDENVNSQP	FMRWRDRFLF	CAEAIYKAQT	ETGEIKGHYL	NATAGTCEEM
IKRAVFAREL	GVPIVMHDYL	TGGFTANTSL	AHYCRDNGLL	LHIHRAMHAV
IDRQKNHGIH	FRVLAKALRM	SGGDHIHSGT	VVGKLEGERD	ITLGFVDLLR
DDFVEQDRSR	GIYFTQDWVS	LPGVLPVASG	GIHVWHMPAL	TEIFGDDSVL
QFGGGTLGHP	WGNAPGAVAN	RLALEACVQA	RNEGRDLAQE	GNEIIREACK
WSPELAAACQ	VWKEIVFNFA	AVDVLDK		
**Spot 6152 (F2-1.mht)**				
MAAAVTAAVS	LPYSNSTSLP	IRTSIVAPER	LVFKKVSLNN	VSISGRVGTI
RAQVTTEAPA	KVVKHSKKQD	ENIVVNKFKP	KEPYVGRCLL	NTKITGDDAP
GETWHMVFST	EGEVPYREGQ	SIGIVPDGID	KNGKPHKLRL	YSIASSAIGD
FGDSKTVSLC	VKRLVYTNDA	GEVVKGVCSN	FLCDLKPGSE	VKITGPVGKE
MLMPKDPNAT	VIMLGTGTGI	APFRSFLWKM	FFEKHEDYQF	NGLAWLFLGV
PTSSSLLYKE	EFEKMKEKAP	ENFRLDFAVS	REQVNDKGEK	MYIQTRMAQY
AEELWELLKK	DNTFVYMCGL	KGMEKGIDDI	MVSLAAKDGI	DWIEYKRTLK
KAEQWNVEVY				
**Spot 6151 (F3-1.mht)**				
MAAAVTAAVS	LPYSNSTSLP	IRTSIVAPER	LVFKKVSLNN	VSISGRVGTI
RAQVTTEAPA	KVVKHSKKQD	ENIVVNKFKP	KEPYVGRCLL	NTKITGDDAP
GETWHMVFST	EGEVPYREGQ	SIGIVPDGID	KNGKPHKLRL	YSIASSAIGD
FGDSKTVSLC	VKRLVYTNDA	GEVVKGVCSN	FLCDLKPGSE	VKITGPVGKE
MLMPKDPNAT	VIMLGTGTGI	APFRSFLWKM	FFEKHEDYQF	NGLAWLFLGV
PTSSSLLYKE	EFEKMKEKAP	ENFRLDFAVS	REQVNDKGEK	MYIQTRMAQY
AEELWELLKK	DNTFVYMCGL	KGMEKGIDDI	MVSLAAKDGI	DWIEYKRTLK
KAEQWNVEVY				
**Spot 2135 (F4-1.mht)**				
PTTTPSTTHP	RRFTVRAARG	KFERKKPHVN	IGTIGHVDHG	KTTLTAALTM
ALASMGNSAP	KKYDEIDAAP	EERARGITIN	TATVEYETEN	RHYAHVDCPG
HADYVKNMIT	GAAQMDGAIL	VVSGADGPMP	QTKEHILLAK	QVGVPNMVVF
LNKQDQVDDE	ELLELVELEV	RELLSSYEFP	GDEIPIISGS	ALLALEALMA
NPSIKRGENQ	WVDKIYQLMD	NVDEYIPIPQ	RQTELPFLMA	IEDVFSITGR
GTVATGRVER	GTVKVGEIVD	IVGLKDTRNT	TVTGVEMFQK	ILDEAMAGDN
VGLLLRGIQK	IDIQRGMVLA	KPGTITPHTK	FEALVYVLKK	EEGGRHSPFF
AGYRPQFYMR	TTDVTGKVTV	IMSDKGEESK	MVMPGDRVNM	VVELIMPVAC
EQGMRFAIRE	GGKTVGAGVI	QKILE		
**Spot 2047 (F5-1.mht)**				
MSPKTETKAS	AGFKAGVKDY	RLTYYTPEYP	TKDTDILAAF	RVTPQPGVPP
EEAGAAVAAE	SSTGTWTTVW	TDGLTSLDRY	KGRCYDIEPV	PGEENQYIVY
VAYPLDLFEE	GSVTNLFTSI	VGNVFGFKAL	RALRLEDLRI	PPAYSKTFQG
PPHGIQVERD	KLNKYGRPLL	GCTIKPKLGL	SAKNYGRAVY	ECLRGGLDFT
KDDENVNSQP	FMRWRDRFCF	CAEALFKAQA	ETGEIKGHYL	NATAGTCEEM
MKRAVFAREL	GVPIVMHDYL	TGGFTANTSL	AHYCRDNGLL	LHIHRAMHAV
IDRQKNHGMH	FRVLAKALRM	SGGDHIHAGT	VVGKLEGERE	VTLGFVDLLR
DDFIEKDRSR	GIYFTQDWVS	MPGVIPVASG	GIHVWHMPAL	TEIFGDDSVL
QFGGGTLGHP	WGNAPGAVAN	RVALEACVEA	RNEGRDLARE	GNEVIREASK
WSPELAAACE	VWKEIIFEFE	TIDTL		

		**Spot 1024 (F14-1.mht)**		
SVGFKAGVKE	YKLTYYTPEY	ETKDTXILAA	FRVTPQPGVP	PEEAGAAVAA
ESSTGTWTTV	WTDGLTSLDR	YKGRCYHIEP	VPGEEDKYIA	YVAYPLDLFE
EGSVTNMFTS	IVGNVFGFKA	LRALRLEDLR	IPPAYIKTFQ	GPPHGIQVER
DKLNKYGRPL	LGCTIKPKLG	LSAKNYGRAV	YECLRGGLDF	TKDDENVNSQ
PFMRWRDRFL	FCAEAIYKAQ	AETGEIKGHY	LNATAGTCEE	MIKRAVFARE
LGVPIVMHDY	LTGGFTANTS	LAHYCRDNGL	LLHIHRAMHA	VIDRQKNHGI
HFRVLAKALR	MSGGDHIHSG	TVVGKLEGER	DITLGFVDLL	RDDFIEKDRS
RGIYFTQDWV	SLPGVLPVAS	GGIHVWHMPA	LTEIFGDDSV	LQFGGGTLGH
PWGNAPGAVA	NRVALEACVQ	ARNEGRDLAA	EGNEIVREAS	KWSPELAAAC
EVWKEITFHF	KAVDTLD			
		**Spot 2042 (F21-1.mht)**		
MSDEEHHFES	KADAGASKTF	PQQAGTIRKN	GYIVIKNRPC	KVMEVSTSKT
GXHGHAKCHF	VGIDIFNGKK	LEDIVPSSHN	CDVPHVNRTD	YQLIDISEDG
FVSLLTETGN	TKDDLRLPTD	ENLLSQIKDG	FAEGKDLVVS	VMSAMGEERI
CSLKDIGPKN				
		**Spot 3026 (G1-1.mht)**		
MAMASTFSLT	NTTITPFLSP	TSPPKISSFR	FSNSRSLPLP	TLSTASSSPT
TISHAPNDYK	DAPIELRYPA	FPSVLDINQI	RNILPHRFPF	LLVDRVIEYN
PGVSAVAIKN	VTINDNFFPG	HFPERPIMPG	VLMVEAMAQV	GGLVMLQPEV
GGSRENFFFA	GIDKVRFRKP	VIAGDTLVMR	MTLIKLQKRF	GIAKMEGKAY
VGGEVVCEGE	FLMATGSGGE			
		**Spot 2137 (G7-1.mht)**		
MALTTKPHHL	QRSFLSPSRV	SGERYLESAP	SCLRFRRSGV	QCSVVAKECR
VKGVKARQII	DSRGNPTVEV	DLITDDLYRS	AVPSGASTGI	YEALELRDGD
KSVYGGKGVL	QAIKNINELV	APKLIGVDVR	NQADVDALML	ELDGTPNKSK
LGANAILGVS	LSVCRAGAGA	KGVPLYKHIQ	ETSGTKELVM	PVPAFNVING
GSHAGNSLAM	QEFMILPVGA	TSFSEAFQMG	SEVYHTLKGI	IKTKYGQDAC
NVGDEGGFAP	NVQDNREGLV	LLIDAIEKAG	YTGKIKIGMD	VAASEFFMKD
GRYDLNFKKQ	PNDGAHVLSA	ESLADLYREF	IKDFPIVSIE	DPFDQDDWSS
WASLQSSVDI	QLVGDDLLVT	NPKRIAEAIK	KQSCNALLLK	VNQIGTVTES
IQAALDSKAA	GWGVMVSHRS	GETEDNFIAD	LSVGLASGQI	KTGAPCRSER
LSKYNQLLRI	EEELGNVRYA	GEAFRSP		
		**Spot 9708 (G8-1.mht)**		
MYKLSSIQCV	APFTISLCTL	SFSSSSLRFS	VRATSSRAMA	SHIVGYPRMG
PKRELKFALE	SFWDGKSSAD	DLQKVAADLR	SAIWKQMADA	GIKYIPSNTF
SYYDQVLDTT	AMLGAVPSRY	NWNGGEIGFD	VYFSMARGNS	SVPAMEMTKW
FDTNYHYIVP	ELGPDVKFSY	ASHKVVDEYK	EAKVLGINTV	PVLVGPVSYL
LLSKPAKGVE	KSFSLLSLID	KILPVYREVV	AELKAAGATW	IQFDEPTLVK
DLNAHQLQAF	THAYAELESS	LSGLNVLIET	YFADVPAEAY	KTLTSLKAVT
AYGFDIVRGT	KTLDLIKQGF	PSGKFLFAGV	VDGRNIWANN	FASSLNTLQA
LGDIVGNDKV	VVSTSCSLLH	TAVDLVNETK	LDQEIKSWLA	FAAQKVVEVN
ALAKALSGQK	DEVFFSANAA	ALASRKSSPR	VINEAVQKAA	AALKGSDHRR
ATNVSARLDA	QQKKLNLSVL	PTTTIGSFPQ	TADLRRVRRE	FKANKISEED
YIRFIKEEIY	NVVKLQEELD	IDVLVHGEPE	RNDMVEYFGE	QLSGFAFTAN
GWVQSYGSRC	VKPPIIYGDV	SRPKPMTVFW	SSTAQSLTKR	PMKGMLTGPV
TILNWSFVRD	DQPRFETCYQ	IALAIKDEVE	DLEKAGITVI	QIDEAALREG

LPLRKSEEAF	YLNWAVHSFR	ITNCGVEDTT	QIHTHMCYSN	FNDIIHSIID
MDADVITIEN	SRSDEKLLSV	FREGVKYGAG	IGPGVYDIHS	PRIPPTEEIA
DRINKMLAVL	ESSILWVNPD	CGLKTRKYTE	VKPALTNMVA	AAKLIRNQL
**Spot 2628 (G13-1.mht)**				
NYAAKDIKFG	VEARALMLKG	VEELADAVKV	TMGPKGRNVV	LEQSFGAPKV
TKDGVTVAKS	IEFRDKVKNI	GASLVKQVAN	ATNDAAGDGT	TCATVLTRAI
FTEGCKSVAA	GMNAMDLRRG	ISMAVDSVVT	NLKSRARMIS	TSEEIAQVGT
ISANGEREIG	ELIAKAMEKV	GKEGVITIAD	GKTLYNELEV	VEGMKLDRGY
ISPYFITNPK	NQKCELEDPL	VLIHEKKISN	LNSIVKILEL	ALKKQRPLLI
VAEDVESEAL	ATLIINKLRA	GIKVCAIKAP	GFGENRKANL	QDLAILTGGE
VITEELGLNL	DKVGVETLGT	CKRVTISKDD	TVILDGAGDK	KAIEERCEQL
RSSIELSTSD	YDKEKLQERL	AKISGGVAVL	KIGGASEAEV	SEKKDRVTDA
LNATKAAVEE	GIVPGGGAAL	LYASKELDKL	ATANFDQKIG	VQIIQNALKM
PVSTIASNAG	VEGAVVVGKL	LEQDNPDLGY	DAAKGEYVDM	IKAGIIDPLK
VIRTALVDAA	SVSSLMTTTE	AVVVELPKDE	KETPGMGGGM	GGMDY
**Spot 2216 (G15-1.mht)**				
SVGFKAGVKE	YKLTYYTPEY	ETKDTXILAA	FRVTPQPGVP	PEEAGAAVAA
ESSTGTWTTV	WTDGLTSLDR	YKGRCYHIEP	VPGEEDKYIA	YVAYPLDLFE
EGSVTNMFTS	IVGNVFGFKA	LRALRLEDLR	IPPAYIKTFQ	GPPHGIQVER
DKLNKYGRPL	LGCTIKPKLG	LSAKNYGRAV	YECLRGGLDF	TKDDENVNSQ
PFMRWRDRFL	FCAEAIYKAQ	AETGEIKGHY	LNATAGTCEE	MIKRAVFARE
LGVPIVMHDY	LTGGFTANTS	LAHYCRDNGL	LLHIHRAMHA	VIDRQKNHGI
HFRVLAKALR	MSGGDHIHSG	TVVGKLEGER	DITLGFVDLL	RDDFIEKDRS
RGIYFTQDWV	SLPGVLPVAS	GGIHVWHMPA	LTEIFGDDSV	LQFGGGTLGH
PWGNAPGAVA	NRVALEACVQ	ARNEGRDLAA	EGNEIVREAS	KWSPELAAAC
EVWKEITFHF	KAVDTLD			
**Spot 0129 (G20-1.mht)**				
MADAKSTIAK	DVTELIGNTP	LVYLNRVVDG	CVARVAAKLE	MMEPCSSVKD
RIGYSMISDA	ENKGLITPGE	SVLIEPTSGN	TGIGLAFIAA	AKGYRLIICM
PASMSLERRT	ILRAFGAELV	LTDPARGMKG	AVQKAEEIKA	KTPNSYILQQ
FENPANPKIH	YETTGPEIWR	GSGGKIDALV	SGIGTGGTVT	GAGKYLKEQN
PNIKLYGVEP	VESAILSGGK	PGPHKIQGIG	AGFIPGVLDV	NLLDEVIQVS
SEESIETAKL	LALKEGLLVG	ISSGAAAAAA	IRIAKRPENA	GKLIVAVFPS
FGERYLSTVL	FESVKRETEN	MVFEP		
**Spot 1227 (H6-1.mht)**				
MKSKKCLAII	LILSTICMTG	CWDKIEINER	AFASVLGVDA	GKDIGKEKQL
KEISDSASFT	GSKFDKIKVT	YAFPDISKLG	PEKGGTAVDN	TMSVDAYSMQ
DSINEVINKS	SRTLSFGHLK	LIVLNTSILD	YSNTFKEVID	YLQRQPAISR
TIYIVFSEDK	SEEILKFKPN	MEKSIENYII	GILENNKKSN	TSSPLTLNEF
LEKTSQNNTT	LMPVINIDKE	NKDLKISKVA	VIKNDKIKGY	ISTKQANSIQ
LINKRFKGGT	RTIIRDGSPL	DYSIENNERK	ISIRDKKKLS	IDINLNLEGQ
IKGYNIDKQI	SSSERNIKEI	EGNLNKAITQ	DMKEVIRISQ	AEYNTDILDI
GEFIHKYHPK	LWKETKGNWN	ELYKTVDINV	SVDTKVRRIG	GIK
**Spot 2128 (H8-1.mht)**				
MSSFINHHFY	PSVCTSKHAL	PINPTSPFYL	GIPNFRQKSR	FMHLTPRCFS
RQIDPLDKQK	KRSFSVKECA	ISLALAAALI	SGVPSLSWER	HAEALTSPVL
PDLAVLISGP	PIKDPEALLR	YALPIDNKAI	REVQKPLEDI	TESLRVLGLK

ALDSVERNLK	QASRALKNGK	SLIIAGLAES	KKDRGVELLD	KLEAGMGELQ
QIVENRNREG	VAPKQRELLQ	YVGSVEEDMV	DGFPYEVPEE	YQTMPLLKGR
AVVEMKVKVK	DNPNVDNCVF	RIVLDGYNAP	VTAGNFLDLV	ERHFYDGMEI
QRRDGFVVQT	GDPEGPAEGF	IDPSTEKPRT	IPLEIMVEGE	KVPVYGSTLE
ELGLYKAQTK	LPFNAFGTMA	MAREEFENNS	GSSQIFWLLK	ESELTPSNAN
ILDGRYAVFG	YVTDNQDYLA	DLKVGDVIES	VQAVSGVDNL	VNPTYKIAG

Spot 2218 (I16-1.mht)

MTQAKLRYAV	VTGANKGIGL	ETVNQLASNG	VKVVLTARDE	DRGHEAIERL
KECGLSDFVX	HQLDVTDSAS	IVSLVEFVKT	QFGRLDILVN	NAGISGVNPY
ETEGSTINWK	ELAQTCEMAE	KCLTTNYYGA	KETTEAFLPL	LQLSNSPRIV
NVSSQAGLLK	VLKEFIKDFK	EGSLKKKGWP	TFLSAYMVSK	AAMNSYTRIL
AKKHQNFCIN	CVCPGFVKTD	INRNTGFLSV	DQGAASVVRN	AVVTGANKGI
GFGICKQLVS	NGITVVLTAR	DEKRGLEAVE	KLKEFGVSDQ	VVFHQLDVTD
PKSIESLANF	IKTQFGKLDI	LVNNAGIHGA	YVDRDALAAA	GSSEKVANVD
WRKISTENFE	AAEAGIRTNY	YGVKLMCEAL	IPLLELSGTP	RIVNVSSSMG
KLEKIPNAWA	RGALSDAESL	TEEKVDEVLN	QFLKDFKEGS	LETKGWPHAF
SAYIVSKAAL	TAYTRILAKK	YPSFCINAVC	PGFVKTDLNY	NTGYLSVDEG
AESVVRLALL	PNGGPSGLFF	SRSEVAPF		

Spot 3024 (I19-1.mht)

MASTTLSPTT	PSQLCSGKSG	IFSSSQALLV	KPVKRQMMGK	SKGLRIACQA
TSISADRVPD	MGKRQLMNLL	LLGAISLPTA	GMLIPYTYFF	VPPGSGSSAG
GTVAKDAVGN	DVIAENWLKA	HGPGDRTLTQ	GLKGGPTYLA	VEKDRTLATF
AINAVCTHLG	CVVPWNQAEN	KFICPCHGSR	YNDQGRVVRG	PAPLSLALAH
CDIDDGKVVF	VPWVETDFRT	GDAPWWA		

Spot 1052 (I21-1.mht)

MASTQCFLHH	QYAITTPTRT	LSQRQVVTTK	PNHIVCKAQK	QDDVVDAVVS
RRLALSVLIG	AAAVGSKVSP	ADAAYGEAAN	VFGKAKTNTD	YLPYNGDGFK
LLVPAKWNPS	KEREFPGQVL	RYEDNFDATS	NVSVLVQTTD	KKSITDYGSP
EEFLSKVDYL	LGKQAFFGQT	DSEGGFDTNA	VAVANILESS	APVIGGKQYY
NISVLTRTAD	GDEGGKHQLI	TATVKDGKLY	ICKAQAGDKR	WFKGARKFVE
DTASSFSVA				

Spot 0137 (I24-1.mht)

MAGSSSLSTL	SLCTQSPSPS	PVASGRLVAP	AVLGFAGAPR	FPTLRAAPRR
LTARAVAGDA	EDEXGKEPAA	DQGGAAAAVA	EAPADVPVTS	EVAELKAKLK
EALYGTERGL	RASSETRAEV	VELITQLEAR	NPTPAPTEAL	TLLNGKWILA
YTSFSQLFPL	LGSGSLPQLV	KVEEISQTID	SENFTVQNCI	KFSGPLATTS
VSTNAKFEVR	SPKRVQIKFD	EGIIGTPQLT	DSIVLPEKFE	LFGQNIDLTP
LKGIFSSIEN	AASSVARTIS	GQPPLKIPIR	TDNAESWLLT	TYLDDELRIS
RGDGSSIFVL	FKEGSTLLY			

Spot 0021 (J14-1.mht)

MAASVSTVGA	VNRAILNLNG	SGAGASAPTS	AFFGTSLKKA	VASRVPNSKV
TNGSFKIVAA	EKEIEESQQT	NKDRWKGLAY	DISDDQQDIT	RGKGMVDPLF
QAPMDAGTHY	AVMSSYEYLS	TGLRQLDNIK	DGFYIAPAFL	DKLVVHITKN
FMTLPNIKVP	LILGIWGGKG	QGKSFQCELV	FAKMGINPIM	MSAGELESGN
AGEPAKLIRQ	RYREAADLIA	KGKMCALFIN	DLDAGAGRLG	GTTQYTVNNQ
MVNATLMNIA	DNPTNVQLPG	MYNKEENARV	PIIVTGNDFS	TLYAPLIRDG
RMEKFYWAPT	RDDRVGVCKG	IFRTDGVPEE	DITKLVDTFP	GQSIDFFGAL

RARVYDDEVR	KWISGVGVDA	TGKKLVNSKE	GPPTFDQPKM	SLDKLLQYGN
MLVQEQENVK	RVQLADKYLN	EAALGNANED	AIKSGSFFK	
**Spot 4015 (J16-1.mht)**				
MACSATSTSF	ISSIAAAKSM	ATPLSKTLTL	PNSFSGTRKS	IQSPVLRSIS
LTRGSHSAKS	FVVKASSELP	LVGNVAPDFE	AEAVFDQEFI	KVKLSEYIGN
KYVVLFFYPL	DFTFVCPTEI	TAFSDRYEEF	KQINTEVLGV	SVDSVFSHLA
WVQTDRKSGG	LGDLKYPLIS	DVTKSISKSY	GVLIPDQGVA	LRGLFIIDKE
GVIQHSTINN	LAIGRSVDET	KRTLQALQYV	QENPDEVCPA	GWKPGDKSMK
PDPRQSKDYF	AAL			
**Spot 1028 (J21-1.mht)**				
MAPIAVGDTI	PEGTLSYFDE	NDQLQQVSVH	SLAAGKKVII	FGVPGAFTPT
CSLKHVPGYV	EKAEELKSKG	VAEVICISVN	DPFVMKAWAK	TFPENKHVKF
LADGSATYTH	ALGLELNLSD	KGLGVRSRRF	AILVEDLKVK	AANIESRGEF
TCSNADDVLK	AL			
**Spot 4115 (K1-1.mht)**				
STLLSDEVLL	NLTDATGKDR	IIAEYIWIGG	SGMDIRSKAR	TLPGPVTDPS
KLPKWNYDGS	STGQAAGEDS	EVILYPQAIF	KDPFRKGNNI	LVMCDAYTPA
GDPIPTNKRH	NAAKIFSHPD	VAKEEPWYGI	EQEYTLMQKD	VNWPIGWPVG
GYPGPQGPYY	CGVGADKAIG	RDIVDAHYKA	CLYAGIGISG	INGEVMPGQW
EFQVGPVEGI	SSGDQVWVAR	YLLERITEIS	GVIVSFDPKP	VPGDWNGAGA
HCNYSTKTMR	NDGGLEVIKK	AIGKLQLKHK	EHIAAYGEGN	ERRLTGKHET
ADINTFSWGV	ANRGASVRVG	RDTEKEGKGY	FEDRRPASNM	DPYVVTSMIA
ETTILGEPTL	EAEALAAQKL	SLNV		
**Spot 1122 (K2-1.mht)**				
MASTSSLTLS	QALLARAISH	HVSTQSSDRL	SLSTPSLPAF	SGLKSTSSSI
PRATSSRRSR	RNSSIPTARR	LQTPTRAAAV	ETLDVTTDTS	LVEKSVNTIR
FLAIDAVEKA	NSGHPGLPMG	CAPMGHILYD	EIMKYNPKNP	YWFNRDRFVL
SAGHGCMLQY	ALLHLAGYDS	VKEEDLKSFR	QWGSKTPGHP	ENFETPGVEV
TTGPLGQGIA	NAVGLALAEK	HLAARFNKPD	NEIVDHYTYA	ILGDGCQMEG
IANEACSLAG	HWGLGKLIAF	YDDNHISIDG	DTEIAFTESV	DKRFEALGWH
VIWVKNGNTG	YDEIRAAIKE	AKAVTDKPTM	IKVTTTIGYG	SPNKANSYSV
HGSALGAKEV	DATRKNLGWP	FEPFHVPEDV	KNHWSRHIPG	GAAFEAEWNA
KFAEYEKKYK	EEAAELKSII	KGELPAGWEK	ALPTYTPESP	ADATRNLSQA
NLNALAKVLP	GLLGGSADLA	SSNMTLLKMF	GDFQKDTPEE	RNVRFGVREH
GMGAICNGIA	LHSPGLIPYC	ATFFVFTDYM	RAAMRISALS	EAGVIYVMTH
DSIGLGEDGP	THQPIEHLAS	FRAMPNILMF	RPADGNETAG	AYRIAVLNRK
RPSVLALSRQ	KLPQLPGTSI	EGVEKGGYTI	SDNSSGNKPD	VILIGTGSEL
EIAAKAAEEL	RREGKAVRVV	SFVSWELFDE	QSDAYKESVL	PAAVTARVSI
EAGSTFGWQK	IVGGKGKAIG	IDRFGASAPA	GKIYKEYGIT	AEAVIAAAKE
LC				
**Spot 6117 (K3-1.mht)**				
MSINFVSIIA	KPKITITTNP	FLVSSPLLFF	SNKTKTKPSI	LTAFSSMSYD
KELAAAKKAA	SLAARLCQKV	QKAILQSDVQ	SKSDKSPVTV	ADYGSQALVS
YVLQRELPSE	LFSLVAEEDS	EDLLKDGGQE	TLERITKLVN	DILATDGSYS
DSTLSTEDIV	KAIDCGKSEG	GSRGRHWVLD	PIDGTKGFLR	GDQYAIALAL
LDEGTVVLGV	LACPNLPITS	IAGGGSHHSL	PGEVGCLFFS	VAGGGTYMHS
LDSSSAVKVQ	VSSIDNPEEA	SFFESYEAAH	SMHDLSSSIA	KKLGVKAPPV

RIDSQAKYGA	LSRGDGVIYL	RFPHKGYREK	IWDHAAGYIV	VAEAGGVVTD
AAGNPLDFSK	GRYLDLDTGI	IVTNQKLMPS	LLKAVRESIE	EKISSL
**Spot 5019 (K4-1.mht)**				
MAQASTAASL	LEWVKKDKRR	MLHVVYRVGD	LDRTIKFYTE	CLGMKLLRKR
DIPEERYANA	FLGYGPEDSH	FVIELTYNYG	VDSYDIGTGF	GHFGIALEDV
AKTVELIKAK	GGKVTREPGP	VKGGSTVIAF	IEDPDGYKFE	LLERGPTPEP
LCQVMLRVGD	LDRSINFYEK	AFGMELLRKR	DNPEYKYTIA	MMGYGPEDKN
AVLELTYNYG	VTEYDKGNAY	AQIAIGTDDV	YRTAEAVKLF	GGKVTREPGP
LPGISTKITA	CLDPDGWKTV	FVDNIDFLKE	LE	
**Spot 6020 (K8-1.mht)**				
MATKEGKAIG	IDLGTTYSCV	GVWQNDRVEI	IPNDQGNRTT	PSYVAFTDTE
RLIGDAAKNQ	VAMNPQNTVF	DAKRLIGRRF	SDSSVQNDMK	LWPFKVGGSP
CDKPMIVVNY	KGEEKKFSAE	EISSMVLVKM	REVAEAFLGH	AVKNAVVTVP
AYFNDSQRQA	TKDAGAISGL	NVLRIINEPT	AAAIAYGLDK	KASRKGEQNV
LIFDLGGGTF	DVSILTIEEG	IFEVKATAGD	THLGGEDFDN	RMVNHFVSEF
KRKNKKDISG	NARALRRLRT	ACERAKRTLS	STAQTTIEID	SLYEGIDFYA
TITRARFEEM	NMDLFRKCME	PVEKCLRDAK	IDKSQVHEVV	LVGGSTRIPK
VHQLLQDFFN	GKELCKSINP	DEAVAYGAAV	QAAILSGQGD	EKVQDLLLLD
VTPLSLGLET	AGGVMTVLIP	RNTTIPTKKE	QIFSTYSDNQ	PGVLIQVFEG
ERARTKDNNL	LGKFELTGIP	PAPRGVPQVN	VCFDIDANGI	LNVSAEDKTA
GVKNKITITN	DKGRLSKEEI	EKMVKDAERY	KAEDEEVKKK	VEAKNSLENY
AYNMRNTIKD	EKIGGKLSPD	EKQKIEKAVE	DAIQWLEGNQ	MAEVDEFEDK
QKELEGICNP	IIAKMYQGAA	GPGGDVPMGA	DMPAAGAGPK	IEEVD
**Spot 2116 (K10-1.mht)**				
METFLFTSES	VNEGHPDKLC	DQVSDAILDA	CLEQDPESKV	ACETCTKTNM
VMVFGEITTK	ASVNYEKIVR	DTCKGIGFVS	PDVGLDADNC	KVLVNIEQQS
PDIAQGVHGH	MTKKPEEIGA	GDQGHMFGYA	TDETPELMPL	THVLATKLGA
RLTEVRKNKT	CPWLRPDGKT	QVTVEYKNDG	GAMIPIRVHT	VLISTQHDET
VTNEQISKEL	KEHVIKPVIP	AKYLDDKTIF	HLNPSGRFVI	GGPHGDAGLT
GRKIIDTYG	GWGAHGGGAF	SGKDPTKVDR	SGAYIVRQAA	KSVVASGLAR
RCIVQVSYAI	GVPEPLSVFV	DTYQTGKIPD	KDILALIKEK	FDFRPGMIAI
HLDLMRGGNF	RYQKTAAYGH	FGREDPDFTW	ETVKILKPNA	
**Spot 1118 (K11-1.mht)**				
MATLQLSDYC	NPVVALKPSL	KSFFIKTDHR	NCCGASRVRV	VPRACLQKPS
LTRRHLLAET	AAISVAPLIL	GIEPPAKSEE	PLLSEWERVY	LPIDPGVVLL
DIAFVPDDLS	HGFLLGTRQT	IMETKDGGNT	WVPRSIPSAE	DEDFNYRFNS
ISFKGKEGWI	VGKPSILLYT	SDAGESWERI	PLSAQLPGDI	VYIKATGEKS
AEMVTDEGAI	YVTSNRGYNW	RAAVQETVSA	TLNRTVSSGI	SGASYYTGTF
NTVNRSPDGS	YVAVSSRGNF	YLTWEPGQPY	WQPHNRAVAR	RIQNMGWRAD
GGLWLLVRGG	GLYLSKGTGI	SEDFEEIPVQ	SRGFGILDVG	YRSKEEAWAA
GGSGVLLRTT	NGGKTWTRDK	AADNIAANLY	SVKFIDDTKG	FVLGNDGVLL
RFLG				
**Spot 1025 (K14-1.mht)**				
MGSSGIDWKL	PDHPKLPKGK	VIGLIVLDGW	GEANADQYNC	IHVAETPVMD
SLKKGAPERW	RLVRAHGTAV	GLPTEDDMGN	SEVGHNALGA	GRIFAQGAKL
VDAALASGKI	FEGEGFKYVK	ESFETNTLHL	IGLMSDGGVH	SRLDQLLLLL
KGASEQGAKK	IRVHILTDGR	DVLDGSSVGF	AEILENDLAK	LREKGVDAQI

ASGGGRMYVT	MDRYENDWSV	VKRGWDAQVL	GEAPYKFKNA	VEAIKTLRAE
PNANDQYLPP	FVIVDDSGKP	VGPIVDGDAV	VTFNFRADRM	VMIAKALEYA
DFDKFDRVRV	PKIRYAGMLQ	YDGELKLPSH	YLVEPPEIDR	TSGEYLTYNG
VRTFACSETV	KFGHVTFFWN	GNRSGYFNDK	MEEYVEIPSD	SGITFNVQPK
MKAVEIAEKA	RDAILSGKFE	QVRVNLPNGD	MVGHTGDIEA	TIVACKAADE
AVKIILDAIE	KVGGIYVVTA	DHGNAEDMVK	RNKTGQPLLD	KSGNIQILTS
HTLQPVPIAI	GGPGLAPGVR	FRKDLPSGGL	ANVAATVMNL	HGFQAPSDYE
PSLIEVVDN				
**Spot 4014 (K16-1.mht)**				
MSSWNSPYYD	TSSYGAGSGG	GGGGGRRSSS	PPRGAGDKKE	TKTKDYQSYT
SNNNNNGSDD	DKDKNKHKIT	SSHKHKDDEK	DRNNHSKDSH	GGGNSSNYNK
DSYGGNSGNP	NNYYGSSTGV	VAGSGSYYGG	GGGYGGGNTS	YGGSLSYGKD
GGYGGSNSPY	GGGSSIIISG	AAPIPHNNFG	GGGTGWPVPP	PPQDGGSGAA
PVFIRAEEVK	VIYHHTPPGH	GSSSSSYSPS	PKYESEGDRR	RSGGAGSGSG
SNSGGGGGGF	FGPAFHAVGG	YIDRKFGLDK	D	
**Spot 5015 (K21-1.mht)**				
ATKKAVAVLK	GTSNVEGVVT	LTQEDDGPTT	VNVRITGLTE	GLHGFHLHEY
GDTTNGCIST	GAHFNPNKLT	HGAPEDEIRH	AGDLGNIIAN	ADGVAEATIV
DTQIPLSGPN	SVVGRALVVH	ELEDDLGKGG	HELSLTTGNA	GGRLACG
**Spot 0038 (K22-1.mht)**				
MAIQSGVSSI	LTYSPVPHRT	PFCKSIKTSI	FANPKISLLY	SSTILDPKMR
PISRNERLTT	FASPEALTVE	DKTSETEEPI	SDDIIEKVEV	AVKQVEKPRL
VLKFIWMEKN	IGLALDQVIP	GHGTIPLSPY	FFWPRKDAWE	ELKTTLESKP
WISQKKMIIL	LNQATDIINL	WQQSGGNLTT		
**Spot 2129 (K24-1.mht)**				
MASHAALAPS	RIPASTRLAS	KASQQYSFLT	QCSFKRLDVA	DFSGLRSSNS
VTFTREASFH	DVIAAQLTTK	PTGAAPVRGE	TVAKLKVAIN	GFGRIGRNFL
RCWHGRKDSP	LDVVVVNDSG	GVKSATHLLK	YDSILGTFKA	DVKIIDNETF
SIDGKPIKVV	SNRDPLKLPW	AELGIDIVIE	GTGVFVDGPG	AGKHIQAGAK
KVIITAPAKG	SDIPTYVVGV	NEKDYGHDVA	NIISNASCTT	NCLAPFVKVL
DEELGIVKGT	MTTTHSYTGD	QRLLDASHRD	LRRARAAALN	IVPTSTGAAK
AVSLVLPQLK	GKLNGIALRV	PTPNVSVVDL	VVNIEKVGVT	AEDVNNAFRK
AAAGPLKGVL	DVCDIPLVSV	DFRCSDFSST	IDSSLTMVMG	GDMVKVVAWY
DNEWGYSQRV	VDLADLVANK	WPGLEGSVAS	GDPLEDFCKD	NPADEECKLY
E				
**Spot 5032 (L1-1.mht)**				
KAGVGFKAGV	KGYKVTYYTP	EYDPKDTDIL	AAFRVTPQPG	VPPEEAGAAV
AAESSTGTWT	TVWTDGLTNL	DRYKGRCYHI	EPVAGEENQF	IAYVAYPLDL
FEEGAVTNMF	TSIVGNVFGF	KALRALRLED	LRIPPAYVKT	FQGPPHGIQV
ERDKLNKYGR	PLLGCTIKPK	LGLSAINPGR	AVYECLRGGL	DFTKDDENVN
SQPFMRWXDR	FLFCAEALYK	AQAETGEIKG	HYLNLTAGTC	EEMIKRAVFA
RELGVPIVMH	DYLTGGFTAN	TSLSHYCRDN	GLLLHIHRAM	HAVIDRQKNH
GIHFRVLAKX	LRMSGGDHIH	SGTVVGKLEG	ERXITLGLVD	LLRDDYIEKD
RSRGIYFTQD	WVSLPGVLPV	ASGGIHVWHM	PALTEIFGDD	SVLQFGGGTL
GHPWENAPGA	VANRVALEAC	VQARNEGRDL	AAEGNEIIRE	ACKWSPELAA
ACEVWKEIKF	EFEAMDTL			
**Spot 2045 (L3-1.mht)**				

MGASTAQIHG	LGAPSFAAAS	MRKSNNVSSR	SVFFGQKLGN	SSAFPAAAFL
NLRSNTSRRN	SSVRPLRIVN	EKVVGIDLGT	TNSAVAAMEG	GKPTIVTNAE
GQRTTPSVVA	YTKNGDRLVG	QIAKRQAVVN	PENTFFSVKR	FIGRKMSEVD
EESKQVSYRV	VRDENGNVKL	ECPAIGKQFA	AEEISAQVLR	KLVDDASKFL
NDKVTKAVVT	VPAYFNDSQR	TATKDAGRIA	GLEVLRIINE	PTAASLAYGF
EKKSNETILV	FDLGGGTFDV	SVLEVGDGVF	EVLSTSGDTH	LGGDDFDKRI
VDWLAANFKR	DEGIDLLKDK	QALQRLTETA	EKAKMELSSL	TQANISLPFI
TATADGPKHI	ETTITRAKFE	ELCSDLLDRL	KTPVENSLRD	AKLSFKDIDE
VILVGGSTRI	PAVQELVKKM	TGKEPNVTVN	PDEVVALGAA	VQAGVLAGDV
SDIVLLDVSP	LSLGLETLGG	VMTKIIPRNT	TLPTSKSEVF	STAADGQTSV
EINVLQGERE	FVRDNKSLGS	FRLDGIPPAP	RGVPQIEVKF	DIDANGILSV
TAIDKGTGKK	QDITITGAST	LPSDEVERMV	SEADKFAKED	KEKRDAIDTK
NQADSVVYQT	EKQLKELGDK	VPGPVKEKVE	SKLGELKEAI	SGGSTEAIKE
AMAALNQEVM	QLGQSLYNQP	GAGAAPGPGA	SSESGPSEST	GKGPEGDVID
ADFSDSK				

Spot 3023 (L4-1.mht)

MSIFEYNGSA	IVAMVGKNCF	AIASDRRLGV	NLQTVATDFQ	RIYKIHDKVF
VGLSGLGTDA	QTLYQRLVFR	HKLYQLREER	DMKPETFASL	VSAILYEKRF
GPYFCQPVIA	GLGDDDKPFI	CTMDFIGAKE	LAKDFVVAGS	ASESLYGACE
ALFKPDMEPE	ELFEVVSQAL	LASVDRDCLS	GWGGHIYIVT	PDEIREKILK
GRMD				

Spot 3056 (L5-1.mht)

MASATAPTTL	SLLKTTASSS	STTRARASLL	PVSGLRPTTL	RRLGFAAADP
AFSHHVASKI	RSFGSGKASR	AVVSMAKKSV	GDLTAADLKG	KKVFVRADLN
VPLDDNQKIT	DDTRIRAAIP	TVKHLIQNGA	KVILSSHLGR	PKGVTPKFSL
APLVPRLSEL	LGIQVVKADD	CIGPEVEKLV	ASLPEGGVLL	LENVRFYKEE
EKNEPEFAKK	LASLADLYVN	DAFGTAHRAH	ASTEGVTKFL	KPSVAGFLLQ
KELDYLVGAV	SSPKRPFAAI	VGGSKVSSKI	GVIESLLEKC	DILLLGGGMI
FTFYKAQGLS	VGSSLVEEDK	LDLATSLLAK	AKLKGVSLLL	PTDVVIADKF
APDANSKVVA	ASAIPDGWMG	LDVGPESVKT	FNEALETTKT	VIWNGPMGVF
EFDKFAVGTE	AIAKKLAELS	GKGVTTIIGG	GDSVAAVEKV	GVAEVMSHIS
TGGGASLELL	EGKELPGVLA	LDEATPVAV		

Spot 0040 (L6-1.mht)

GLDIAVRLLE	PIKEQFPILS	YADFYQLAGV	VAVEITGGPD	VPFHPGRPDK
KESPPEGRLP	DATKGNDHLR	AVFGHMGLSD	KDIVALSGAH	TLGRCHKERS
GFEGPWTTNP	LIFDNSYFKE	LLSGEKEGLI	QLPSDKALLE	DPVFRPLVEK
YAA				

Spot 4029 (L12-1.mht)

MQNMSLIASL	SSTTSQLTSL	PSLPSSTSPL	SRKFPLTFRE	KNRTPITPHK
LPFSARPTTT	RVVLVKSQAT	APASSEAVAV	TPVSSEMKAW	VYGEYGGVDV
LKLDSNVTVP	DVKEDQVLIK	VVAAALNPVD	AKRRQGKFKA	TDSPLPTVPG
YDVAGVVVKV	GSQVKDFKVG	DEVYGDVNEK	ALEGPKQFGS	LAEYTAVEEK
LLASKPKNLD	FAQAAALPLA	IETAYEGLER	TGFSPGKSIL	VLNGSGGVGS
LVIQLAKQVF	GASRVAATSS	TRNLDLLKSL	GADLAIDYTK	ENFEDLPEKF
DVVYDAIGQC	DRAVKAVKEG	GSVVALTGAV	TPPGFRFVVT	SNGAVLRKLN
PYLESGKVKP	IVDPKGPFSF	DKLAEAFSYL	ETNRATGKVV	IHPIP

Spot 3047 (L15-1.mht)

KSAENNLYVV	EGMQFDRGYI	SPYFVTDSEK	MSVEYENCKL	LLVDKKITNA
RDMITVLEDA	IRGGYPILII	AEDIEQEALA	TLVVN	
**Spot 4026 (L20-1.mht)**				
MAAIPHTNPS	ITTKPPSSPP	RPTFLARFTF	PITSTSHKRH	RLHISNVLSD
SKPTITHSPL	PTESFISRYA	PDQPRKGADV	LVEALEREGV	TDVFAYPGGA
SMEIHQALTR	STTIRNVLPR	HEQGGVFAAE	GYARASGLPG	VCIATSGPGA
TNLVSGLADA	LLDSVPMVAI	TGQVPRRMIG	TDAFQETPIV	EVTRSITKHN
YLVLDVEDIP	RIVREAFYLA	SSGRPGPVLI	DVPKDIQQQL	VVPKWDEPIR
LPGYLSRLPK	TENNGQLEHI	VRLVSEAKRP	VLYVGGGCLN	SGDELRRFVE
LTGIPVASTL	MGLGAYPASS	DLSLHMLGMH	GTVYANYAVD	KSDLLLAFGV
RFDDRVTGKL	EAFASRAKIV	HIDIDSAEIG	KNKQPHVSIC	GDIKVALQGL
NKILEVKNSV	TNLDFSNWRK	ELDEQKVKYP	LSFKTFGEAI	PPQYAIQVLD
ELTGGNAIIS	TGVGQHQMWA	AQFYKYNKPR	QWLTSGGLGA	MGFGLPAAIG
AAVARPDAVV	VDIDGDGSFI	MSVQELATIR	VENLPVKILL	LNNQHLGMVV
QLEDRFYKAN	RAHTYLGNPS	KESEIFPNML	KFAEACDIPA	ARVTRKADLR
AAIQKMLDTP	GPYLLDVIVP	HQEHVLPMIP	AGGGFMDVIT	EGDGRMKY
**Spot 2046 (M3-1.mht)**				
MAAATSSIAP	SLSCPSPSSS	SKTLWSSKAR	TLALPNIGFL	SSSSKSLRSL
TATVAGNGAT	GSSLAARMVS	SSAVKAPVSL	DFETSVFKKE	KVSLAGYEEY
IVRGGRDLFK	HLPDAFKGIK	QIGVIGWGSQ	GPAQAQNLRD	SLVEAKSDIV
VKIGLRKGSR	SFEEARAAGF	TEESGTLGDI	WETIAGSDLV	LLLISDAAQA
DNYEKIFSHM	KPNSILGLSH	GFLLGHLQSS	GLDFPKNISV	VAVCPKGMGP
SVRRLYVQGK	EINGAGINAS	FAVHQDVDGR	AADVRLGWSV	ALGSPFTFAT
TLEQEYRSDI	FGERGILLGA	VHGIVESLFR	RYTENGMSED	LAYKNTVECI
TGTISRTIST	QGMLAVYNSL	SEEGKKDFET	AYSASFYPCM	EILYECYEDV
QSGSEIRSVV	LAGRRFYEKE	GLPAFPMGNI	DQTRMWKVGE	RVRKSRPAGD
LGPLYPFTAG	VYVALMMAQI	EILRKKGHSY	SEIINESVIE	SVDSLNPFMH
ARGVSFMVDN	CSTTARLGSR	KWAPRFDYIL	TQQALVAVDS	GAAINRDLIS
NFFSDPVHGA	IEVCAQLRPT	VDISVPADVD	FVRPELRQSS	N
**Spot 5033 (M4-1.mht)**				
MATAALLRSL	RRREFATSSI	SAYRTLASNT	KPSWCPSLVG	AKWAGLARPF
SSKPAGNEII	GIDLGTTNSC	VAVMEGKNPK	VIENSEGART	TPSVVAFNQK
GELLVGTPAK	RQAVTNPTNT	LSGTKRLIGR	RFDDPQTQKE	MKMVPYKIVR
GSNGDAWVEA	NGQQYSPTQI	GAFILTKMKE	TAEAYLGKSI	NKAVITVPAY
FNDAQRQAIK	DAGAIAGLDV	QRIINEPTAA	ALSYGMNSKE	GLVAVFDLGG
GTFDVSILEI	SNGVFEVKAT	NGDTFLGGED	FDNALLEFLV	SEFKRTEGID
LSKDKLALQR	LREAAEKAKI	ELSSTSQTDI	NLPFITADAS	GAKHLNITLT
RSKFETLVNH	LIERTRNPCK	NCLKDAGVSL	KDVDEVLLVG	GMTRVPKVQE
IVSEIFGKSP	SKGVNPDEAV	AMGAALQGGI	LRGDVKELLL	LDVTPLARGI
ETLGGIFTRL	INRNTTIPTK	KSQVFSTAAD	NQTQVGIKVL	QGEREMASDN
KLLGEFDLVG	IPPAPKGYCP	QIEVIFDIDA	NGMVTVSAKD	KATSKEQQIT
IRSSGGLSED	EIDKMVREAE	MHAQRIKNAR	HLLISGIVQS	TTIYSIEKSL
SEYKEKVPKE	VVTEIETAIS	DLRAAMGTEN	IDDIKAKLDA	ANKAVSKIGE
HMAGGSSGGA	SGGGGAQGGD	QPPEAEYEEV	KK	
**Spot 0041 (M6-1.mht)**				
MALLQNPFLV	QRLPSHTGIA	HARSKGGLSK	FRAAPSTVAL	FELKPPPYSF
DALEPHMSKR	TFEFHWGKHH	RAYVDNLNKQ	IEGTELEGYT	LGDVIKATYS

NGEPQPAFNN	AAQAWNHEFF	WECMSPGGGK	KPGGEILKLI	EKDFGSYDEF
LKEFKQAAAT	QFGSGWAWLV	LKDKKLVVEK	SPNAINPLIW	GHTPLLTIDV
WEHAYYLDYQ	NRRPDYISVF	MNELVSWDGV	NARLDRANA	
**Spot 2048 (M8-1.mht)**				
MAQILAPSTQ	WQMRMTKNPA	NASPFTTKMW	GSLFLKQKTK	GTAKFSTKFR
VCASKSESST	VNRLDDLLNM	DIRPYTDKII	AEYVWIGGTG	IDLRSKSRTI
SKPVEHPSEL	PKWNYDGSST	GQAPGEDSEV	ILYPQAIFKD	PFRGGNNILV
ICDSYTPAGE	PIPTNKRHRA	AEIFGNKKVI	DEVPWFGIEQ	EYTLLQPNVK
WPLGWPVGAY	PGPQGPYYCA	AGADKSFGRD	ISDAHYKACL	YAGINISGTN
GEVMPGQWEY	QVGPSVGIEA	GDHIWCSRYI	LERITEQAGV	VLTLDPKPIE
GDWNGAGCHT	NYSTKSMRED	GGYELIKKAI	LNLSLRHKEH	ISAYGEGNER
RLTGKHETAN	INTFSWGVAN	RGCSVRVGRD	TEKQGKGYLE	DRRPASNMDP
YIVTSLLAET	TILWEPTLEA	EALAAQKLAL	KV	
**Spot 3939 (M10-1.mht)**				
MARALVQSTS	IPSSVAGERT	TKFNGSGKTK	RAVTMLCNAQ	SSSLTLRDFT
GLRGCNAIDT	LVRSGETLQS	KVAAATYVRR	PRGCRFVPKA	MFERFTEKAI
KVIMLAQEEA	RRLGHNFVGT	EQILLGLIGE	GTGIAAKVLK	SMGINLKDAR
VEVEKIIGRG	SGFVAVEIPF	TPRAKRVLEL	SLEEARQLGH	NYIGSEHLLL
GLLREGEGVA	ARVLENLGAD	PSNIRTQVIR	MVGESNEAVG	ASVGGGTSGQ
KMPTLEEYGT	NLTKLAEEGK	LDPVVGRQPQ	IERVTQILGR	RTKNNPCLIG
EPGVGKTAIA	EGLAQRIANG	DVPETIEGKK	VITLDMGLLV	AGTKYRGEFE
ERLKKLMEEI	KQSDEIILFI	DEVHTLIGAG	AAEGAIDAAN	ILKPALARGE
LQCIGATTLD	EYRKHIEKDP	ALERRFQPVK	VPEPTVDETI	QILKGLRERY
EIHHKLRYTD	EDLVAAAQLS	YQYISDRFLP	DKAIDLIDEA	GSRVRLRHAQ
LPEEAKELEK	ELRQITKEKN	EAVRGQDFEK	AGELRDREMD	LKAQITALID
KNKEVSKAES	EAADTGPLVT	EADIQHIVSS	WTGIPVEKVS	TDESDRLLKM
EETLHTRIIG	QDEAVKAISR	AIRRARVGLK	NPNRPIASFI	FSGPTGVGKS
ELAKALAAYY	FGSEEAMIRL	DMSEFMERHT	VSKLIGSPPG	YVGYTEGGQL
TEAVRRRPYT	VVLFDEIEKA	HPDVFNMMLQ	ILEDGRLTDS	KGRTVDFKNT
LLIMTSNVGS	SVIEKGGRRI	GFDLDLDEKD	SSYNRIKSLV	TEELKQYFRP
EFLNRLDEMI	VFRQLTKLEV	KEIADIMLKE	VFERLKVKEI	ELQVTERFRD
RVVDEGYNPS	YGARPLRRAI	MRLLEDSMAE	KMLANEIKEG	DSVIVDVDSD
GNVTVLNGSS	GTPSDPAPEP	IPV		
**Spot 1054 (M11-1.mht)**				
QDPDSKVACE	TCTKTNMVMV	FGEITTKGNI	DYEKIVRDTC	RNIGFVSDDV
GLDADNCKVL	VNIEQQSPDI	AQGVHGHLTK	RPEEIGAGDQ	GHMFGYATDE
TPELMPLSHV	LATKLGARLT	EVRKDGTCPW	LRPDGKTQVT	IEYYNENGAM
VPIRVHTVLI	STQHDETVTN	DKIAADLKEH	VIRPVIPEKY	LDEKTIFHLN
PSGRFVIGGP	HGDAGLTGRK	IIIDTYGGWG	AHGGGAFSGK	DPTKVDRSGA
YIVRQAAKSI	VASGLARRCI	VQVSYAIGVP	EPLSVFVDTY	GTGKIPDKEI
LKIVKESFDF	RPGMIAIHLD	LKRGGNGRFL	KTAAYGHFGR	DDADFTWEVV
KPLKWEKPQD				
**Spot 2239 (M12-1.mht)**				
MAGAAAAVAS	GISIRPVAAP	KISRAPRSRS	VVRAAVSIDE	KAYTVQKSEE
IFNAAKELMP	GGVNSPVRAF	KSVGGQPIVF	DSVKGSHMWD	VDGNEYIDYV
GSWGPAIIGH	ADDKVNAALI	ETLKKGTSFG	APCALENVLA	QMVISAVPSI
EMVRFVNSGT	EACMGALRLV	RAFTGREKIL	KFEGCYHGHA	DSFLVKAGSG

VATLGLPDSP	GVPKGATVGT	LTAPYNDADA	VKKLFEDNKG	EIAAVFLEPV
VGNAGFIPPQ	PAFLNALREV	TKQDGALLVF	DEVMTGFRLA	YGGAQEYFGI
TPDVTTLGKI	IGGGLPVGAY	GGRKDIMEMV	APAGPMYQAG	TLSGNPLAMT
AGIHTLKRLM	EPGTYEYLDK	VTGELVRGIL	DVGAKTGHEM	CGGHIRGMFG
FFFAGGPVHN	FDDAKKSDTA	KFGRFHRGML	GEGVYLAPSQ	FEAGFTSLAH
TTQDIEKTVE	AAEKVLRWI			
**Spot 3055 (M15-1.mht)**				
MAASSACLLG	NGLSVYTTKQ	RFQKLGLDRT	SKVTVVKASL	DEKKHEGRRG
FFKLLLGNAA	AGVGLLASGN	ANADEQGQGV	SSSRMSYSRF	LEYLDKGRVE
KVDLYENGTI	AIVEAVSPEL	GNRIQRVRVQ	LPGLSQELLQ	KLRAKNIDFA
AHNAQEDQGS	PILNLIGNLA	FPVILIGGLF	LLSRRSSGGM	GGPGGPGFPL
QIGQSKAKFQ	MEPNTGVTFD	DVAGVDEAKQ	DFMEVVEFLK	KPERFTAVGA
RIPKGVLLVG	PPGTGKTLLA	KAIAGEAGVP	FFSISGSEFV	EMFVGVGASR
VRDLFKKAKE	NAPCIVFVDE	IDAVGRQRGT	GIGGGNDERE	QTLNQLLTEM
DGFEGNTGVI	VVAATNRADI	LDSALLRPGR	FDRQVSVDVP	DVKGRTDILK
VHSGNKKFES	GVSLEVIAMR	TPGFSGADLA	NLLNEAAILA	GRRGKTAISS
KEIDDSIDRI	VAGMEGTVMT	DGKSKSLVAY	HEVGHAICGT	LTPGHDAVQK
VTLIPRGQAR	GLTWFIPSDD	PTLISKQQLF	ARIVGGLGGR	AAEEVIFGES
EVTTGAVSDL	QQITGLAKQM	VTTFGMSEIG	PWSLMDSSEQ	SDVIMRMMAR
NSMSEKLAND	IDTAVKTLSD	KAYEIALSQI	RNNREAMDKI	VEILLEKETM
SGDEFRAILS	EF			
**Spot 2240 (M16-1.mht)**				
KYFNFHSKRK	CIITQSTLNK	KPNSDNFKNA	QSKAALAALL	FSSITPHAIA
LDDAAPIASP	PQVMEVEAPN	PNTSNPLPFS	QNLVLNAPKT	QASPVSDLPE
STQWRYSEFL	NAVKKGKVER	VRFSKDGSAL	QLTAVDGRRA	NVIVPNDPDL
IDILAMNGVD	ISVSEGEGGN	GLFSVIGNLL	FPFIAFAGLF	FLFRRSQGGP
GGPGGLGGPM	DFGRSKSKFQ	EVPETGVTFA	DVAGADQAKL	ELQEVVDFLK
NPDKYTALGA	KIPKGCLLVG	PPGTGKTLLA	RAVAGEAGVP	FFSCAASEFV
ELFVGVGASR	VRHLFENAKS	KAPCIVFIDE	IDAVGRQRGA	GLGGGNDERE
QTINQLLTEM	DGFSGNSGVI	VLAATNRPDV	LDSALLRPGK	FDRQVTVDRP
DVAGRVRILQ	VHSRGKALAK	DVDFDKIARR	TPGFTGADLQ	NLMNEAAILA
ARRDLKEISK	DEISDALERI	IAGPEKKNAV	VSDEKKKLVA	YHEAGHALVG
ALMPEYDPVA	KISIIPRGQA	GGLTFFAPSE	ERLESGLYSR	SYLENQMAVA
LGGRVAEEVI	FGEDNVTTGA	SNDFMQVSRV	ARQMVERLGF	SKKIGQVAIG
GGGGNPFLGQ	QMSTQKDYSM	ATADVVDSEV	RELVEKAYER	AKQIITTHID
ILHKLAQLLI	EK			
**Spot 4022 (M17-1.mht)**				
MASMASIGSL	KVPSSPSTAT	TSSNSNNHSR	RSVVKRLAFS	SSQLSGDKIF
SKAVTGDRRS	ERRPIVVSPQ	AVSDSKNSQT	CLDPEASRSV	LGIILGGGAG
TRLYPLTKKR	AKPAVPLGAN	YRLIDIPVSN	CLNSNISKIY	VLTQFNSASL
NRHLSRAYAS	NMGGYKNEGF	VEVLAAQQSP	ENPNWFQGTA	DAVRQYLWLF
EEHNVLEFLV	LAGDHLYRMD	YERFIQAHRE	TDADITVAAL	PMDEKRATAF
GLMKIDEEGR	IIEFSEKPKG	EQLKAMKVDT	TILGLDDERA	KEMPYIASMG
IYVISKDVML	NLLRDKFPGA	NDFGSEVIPG	ATSIGMRVQA	YLYDGYWEDI
GTIEAFYNAN	LGITKKPIPD	FSFYDRSAPI	YTQPRYLPPS	KMLDADVTDS
VIGEGCVIKN	CKIHHSVVGL	RSCISEGAII	EDTLLMGADY	YETDADRRFL
AAKGSVPIGI	GKNSHIKRAI	IDKDARIGDN	VKIVNSDSVQ	EAARETDGYF

IKSGIVTIIK	DALIPSGTII			
**Spot 0039 (M20-1.mht)**				
MAAEVPVRAP	AAAARSARSR	PAAAAAVVSA	SSASRLLLGY	RPFHAPRFAA
GRAAVAGPVA	GLRPRSRRPR	LSVVAMAGSD	RQVPLHDYRN	IGIMAHIDAG
KTTTTERILY	YTGRNYKIGE	VHEGTATMDW	MEQEQERGIT	ITSAATTAFW
NKHRINIIDT	PGHVDFTLEV	ERALRVLDGA	ICLFDSVAGV	EPQSETVWRQ
ADKYGVPRIC	FVNKMDRLGA	NFFRTRDMIV	ANLGAKPLVI	QLPIGSEDNF
QGVIDLVRMK	AIVWTGEELG	AKFEYKDIPD	DLQELAQDYR	VQMLETIIEL
DDEVMENYLE	GTEPDEETVK	KLIRKGTISA	SFVPVLCGSA	FKNKGVQPLL
DAVVDYLPSP	LDLPSMKGTD	PEDPEIIFER	QPSDDEPFSG	LAFKIMTDPF
VGSLTFVRIY	SGKLVAGSYV	LNANKDKKER	IGRLLEMHAN	SKEDIPVAVT
GDIVALAGLK	DTITGETLCD	PDKPVVLERM	EFPDPVIKVA	IEPKTKADAD
KMANGLIKLA	QEDPSFHFSR	DEETNQTVIE	GMGELHLDII	VDRLKREFKV
EANVGAPQVN	YRESISKVAE	IQYVHKKQSG	GSGQFADIIV	RFEPLEAGSG
YEFKSEIKGG	AVPKEYVPGV	MKGLEESLPN	GVLAGYPVVD	FRAVLVDGSY
HDVDSSVLAF	QIAARGAFRE	GMRKAGPRLL	EPIMRVEVIT	PEEHLGDVIG
DLNSRRGQVN	SFGDKPGGLK	VVDAFVPLAE	MFQYVSTLRG	MTKGRASYTM
QLAKFDVVPQ	HIQNQLSTKT	EEATA		
**Spot 3048 (M21-1.mht)**				
MSLLSDLINL	NLSDTTEKVI	AEYIWIGGSG	LDLRSKARTL	PGPVKNPSEL
PKWNYDGSST	GQAPGQDSEV	IIYPQAIFKD	PFRRGNNILV	ICDAYTPAGE
PIPTNKRHNA	AKIFSNPDVV	AEEPWYGIEQ	EYTLLQKEVN	WPVGWPVGGF
PGPQGPYYCG	VGADKAFGRD	IVDAHYKACV	YAGINISGIN	GEVMPGQWEF
QVGPAVGISA	GDELWVARYI	LERITEVAGV	VLSFDPKPIK	GDWNGAGAHT
NYSTKTMRND	GGYEEIKSAI	QKLGKRHKEH	IAAYGEGNER	RLTGRHETAD
INTFLWGVAN	RGASIRVGRD	TEKAGKGYFE	DRRPASNMDP	YVVTSMIADT
TILWKP				
**Spot 4031 (N1-1.mht)**				
MAASSACLLG	NGLSVYTTKQ	RFQKLGLDRT	SKVTVVKASL	DEKKHEGRRG
FFKLLLGNAA	AGVGLLASGN	ANADEQGQGV	SSSRMSYSRF	LEYLDKGRVE
KVDLYENGTI	AIVEAVSPEL	GNRIQRVRVQ	LPGLSQELLQ	KLRAKNIDFA
AHNAQEDQGS	PILNLIGNLA	FPVILIGGLF	LLSRRSSGGM	GGPGGPGFPL
QIGQSKAKFQ	MEPNTGVTFD	DVAGVDEAKQ	DFMEVVEFLK	KPERFTAVGA
RIPKGVLLVG	PPGTGKTLLA	KAIAGEAGVP	FFSISGSEFV	EMFVGVGASR
VRDLFKKAKE	NAPCIVFVDE	IDAVGRQRGT	GIGGGNDERE	QTLNQLLTEM
DGFEGNTGVI	VVAATNRADI	LDSALLRPGR	FDRQVSVDVP	DVKGRTDILK
VHSGNKKFES	GVSLEVIAMR	TPGFSGADLA	NLLNEAAILA	GRRGKTAISS
KEIDDSIDRI	VAGMEGTVMT	DGKSKSLVAY	HEVGHAICGT	LTPGHDAVQK
VTLIPRGQAR	GLTWFIPSDD	PTLISKQQLF	ARIVGGLGGR	AAEEVIFGES
EVTTGAVSDL	QQITGLAKQM	VTTFGMSEIG	PWSLMDSSEQ	SDVIMRMMAR
NSMSEKLAND	IDTAVKTLSD	KAYEIALSQI	RNNREAMDKI	VEILLEKETM
SGDEFRAILS	EFTEIPPENR	VASSTSTSTP	TPASV	
**Spot 3049 (N4-1.mht)**				
MATVTASSNF	VSRTSLFNNH	GASSCSDVAQ	ITLKGQSLTH	CGLRSFNMVD
NLQRRSQAKP	VSAKSSKRSS	KVKTAGKIVC	EKGMSVIFIG	AEVGPWSKTG
GLGDVLGGLP	PALAARGHRV	MTICPRYDQY	KDAWDTCVVV	QIKVGDKVEN
VRFFHCYKRG	VDRVFVDHPI	FLAKVVGKTG	SKIYGPITGV	DYNDNQLRFS

LLCQAALEAP	QVLNLNSSKY	FSGPYGEDVV	FVANDWHTAL	LPCYLKSMYQ
SRGVYMNAKV	VFCIHNIAYQ	GRFAFDDYSL	LNLPISFKSS	FDFMDGYEKP
VKGRKINWMK	AAILEAHRVL	TVSPYYAQEL	ISGVDRGVEL	HKYLRMKTVS
GIINGMDVQE	WNPSTDKYID	IKYDITTVTD	AKPLIKEALQ	AAVGLPVDRD
VPVIGFIGRL	EEQKGSDILV	EAISKFMGLN	VQMVILGTGK	KKMEAQILEL
EEKFPGKAVG	VAKFNVPLAH	MITAGADFII	VPSRFEPCGL	IQLHAMRYGT
VPIVASTGGL	VDTVKDGYTG	FHIGRFNVKC	EVVDPDDVIA	TAKAVTRAVA
VYGTSAMQEM	VKNCMDQDFS	WKGPARLWEK	VLLSLNVAGS	EAGTEGEEIA
PLAKENVATP				
		**Spot 3050 (N6-1.mht)**		
MASTAATAAL	SIIKSTGGAA	VTRSSRASFG	HIPSTSVSAR	RLGFSAVVDS
RFSVHVASKV	HSVRGKGARG	VITMAKKSVG	DLNSVDLKGK	KVFVRADLNV
PLDDNQNITD	DTRIRAAIPT	IKFLIENGAK	VILSTHLGRP	KGVTPKFSLA
PLVPRLSELL	GIEVVKADDC	IGPEVETLVA	SLPEGGVLLL	ENVRFYKEEE
KNEPDFAKKL	ASLADLYVND	AFGTAHRAHA	STEGVTKFLK	PSVAGFLLQK
ELDYLVGAVS	NPKRPFAAIV	GGSKVSSKIG	VIESLLEKCD	ILLLGGGMIF
TFYKAQGLSV	GSSLVEEDKL	ELATTLLAKA	KARGVSLLLP	TDVVIADKFA
PDANSKIVPA	SAIPDGWMGL	DIGPDSVKTF	NEALDTTQTV	IWNGPMGVFE
FEKFAKGTEA	VANKLAELSK	KGVTTIIGGG	DSVAAVEKVG	VAGVMSHIST
GGGASLELLE	GKVLPGVVAL	DEATPVTV		
		**Spot 3051 (N12-1.mht)**		
MATITASHFV	SHVCGGATSG	ESKVGLGQLA	LRSQAVTHNG	LRPVNKIDML
QLRTSARNLA	KMEGKMRVEW	QAGTIVCKQQ	GMNLVFVGCE	EGPWCKTGGL
GDVLGGLPPA	LAARGHRVMT	VCPRYDQYKD	AWETCVVVEP	QVGDRIEPVR
FFHSYKRGVD	RVFVDHPMFL	EKVWGKTGSM	LYGPKAGKDY	KDNQLRFSLL
CQAALEAPRV	LNLNSSKYFS	GPYGEDVVFV	ANDWHTALLP	CYLKTMYQSR
GIYMNAKVAF	CIHNIAYQGR	FAFSDFSLLN	LPDEYKGSFD	FIDGYDKPVK
GRKINWMKAG	IREADRVFTV	SPNYAKELVS	CVSKGVELDN	HIRDCGITGI
CNGMDTQEWN	PATDKYLAVK	YDITTVMQAK	PLLKEALQAA	VGLPVDRNIP
LIGFIGRLEE	QKGSDILYAA	ISKFISMDVQ	ILILGTGKKK	FEQQIEQLEV
MYPDKARGVA	KFNVPLAHMI	TAGADFMLIP	SRFEPCGLIQ	LHAMRYGTPC
ICASTGGLVD	TVKEGYTGFH	MGAFNVDCET	VDPEDVLKVI	TTVGRALAIY
GTLAFTEMIK	NCMSQELSWK	GPAKNWETVL	LSLGVAGSEP	GVEGEEIAPL
AKENVATP				
		**Spot 1048 (N13-1.mht)**		
MGSTGEIKYG	AYTYENLERE	PYWPSEKLRI	SITGAGGFIA	SHIARRLKNE
GHYIIASDWK	KNEHMTEDMF	CHEFHLADLR	VMDNCLKVTK	NVDHVFNLAA
DMGGMGFIQS	NHSVIFYNNT	MISFNMVGAA	RINDVKRFFY	ASSACIYPEF
KQLETNVSLK	ESDARPAEPQ	DAYGLEKLAT	EELCKHYTKD	FGIECRIGRF
HNIYGPFGAW	KGGREKAPAA	FCRKTLTATD	KFEMWGDGLQ	TRSFTFIDEC
VEGVLRLTKS	DFREPVNIGS	DEMVSMNEMA	EIVLSFEDKK	LPIQHIPGPE
GVRGRNSDNT	LIKEKLGWAP	TMRLKDGLRI	TYFWIKEQIE	KEKAQGADLS
VYGSSKVVGT	QAPVQLGSLR	AADGKE		
		**Spot 5031 (N17-1.mht)**		
MATIKAVKAR	QIFDSRGNPT	VEVDVILSDG	SFHRAAVPSG	ASTGVYEALE
LRDGGSDYLG	KGVLKAVENV	NSIIAPALLG	KDPTKQTEID	NFMVQQLDGT
VNEWGWCKQK	LGANAILAVS	LAVCKAGAAV	KKIPLYKHIA	NLAGNKTLVL

PVPSFNVING	GSHAGNKLAM	QEFMILPVGA	SSFKEAMKMG	VEVYHHLKAV
IKKKYGQDAT	NVGDEGGFAP	NIQENQEGLE	LLKTAIAKAG	YTGKVVIGMD
VAASEFYDNK	DKTYDLNFKE	ENNDGSQKIS	GDSLKNVYKS	YVTDYPIVSI
EDPFDQDDWE	HYAKLTAEVG	QQVQIVGDDL	LVTNPKRVEK	AIKEKACNAL
LLKVNQIGSV	TESIEAVRMS	KQAGWGVMAS	HRSGETEDTF	IADLSVGLAT
GQIKTGAPCR	SERLAKYNQL	LRIEEELGSA	AVYAGAKFRA	PVEPY
**Spot 1049 (N18-1.mht)**				
MAKPISIEVW	NPSGKYRVVS	TKSMPGTRWI	RLLTDNDCRL	EICTEKKTIL
SVDDILALIG	DHCHGVIGQL	TEDWGEVLFS	ALKRAGGTAF	SNMAVGYNNV
DVDAANRNGI	AIGNTPGVLT	ETTAELAASL	SVAAARRIVE	ADQFMRAGLY
DGWLPHLFVG	NLLKGQTVGV	IGAGRIGSAY	ARMMIEGFKM	NLIYYDLYQS
TRLEKFVTAY	GQFLKANGEQ	GVTWKRAGSM	EEVLREADVI	SLHPVLDKTT
YHLINPERLA	MMKKEAVLVN	ASRGPVIDEA	ALVEHLKANP	MFRVGLDVFE
DEPYMKPGLA	EMKNAVVVPH	IASASKWTRE	GMATLAALNV	LGKIKGYPVW
GNPNAVEPFL	DENATPPPAC	PSIVNAKQLG	LPSSKL	

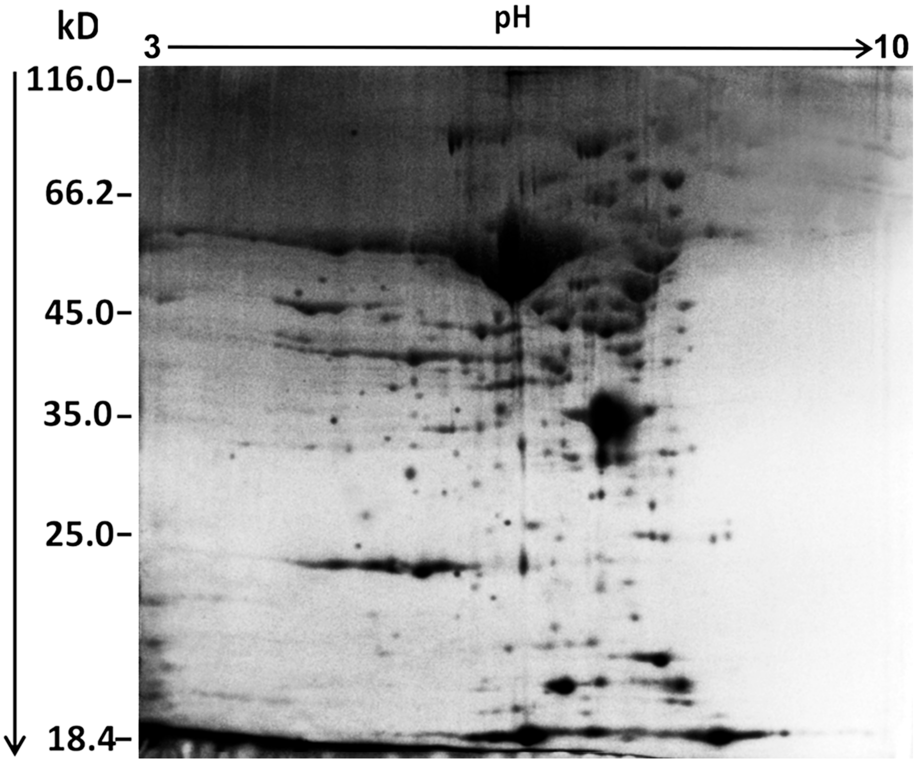

Figure S1. 2-DE profile of total proteins with IPG strips of pH 3–10 in *G. biloba*.
